# Human discrimination and modeling of high-frequency complex tones shed light on the neural codes for pitch

**DOI:** 10.1371/journal.pcbi.1009889

**Published:** 2022-03-03

**Authors:** Daniel R. Guest, Andrew J. Oxenham

**Affiliations:** Department of Psychology, University of Minnesota, Minneapolis, Minnesota, United States of America; University of California at Berkeley, UNITED STATES

## Abstract

Accurate pitch perception of harmonic complex tones is widely believed to rely on temporal fine structure information conveyed by the precise phase-locked responses of auditory-nerve fibers. However, accurate pitch perception remains possible even when spectrally resolved harmonics are presented at frequencies beyond the putative limits of neural phase locking, and it is unclear whether residual temporal information, or a coarser rate-place code, underlies this ability. We addressed this question by measuring human pitch discrimination at low and high frequencies for harmonic complex tones, presented either in isolation or in the presence of concurrent complex-tone maskers. We found that concurrent complex-tone maskers impaired performance at both low and high frequencies, although the impairment introduced by adding maskers at high frequencies relative to low frequencies differed between the tested masker types. We then combined simulated auditory-nerve responses to our stimuli with ideal-observer analysis to quantify the extent to which performance was limited by peripheral factors. We found that the worsening of both frequency discrimination and F0 discrimination at high frequencies could be well accounted for (in relative terms) by optimal decoding of all available information at the level of the auditory nerve. A Python package is provided to reproduce these results, and to simulate responses to acoustic stimuli from the three previously published models of the human auditory nerve used in our analyses.

## Author summary

Pitch, the quality of sound that distinguishes “low” sounds from “high” sounds, is of critical importance for human hearing. In addition to the role of pitch in defining musical melodies and harmony, the pitch of the human voice helps us identify talkers, attend to a particular talker in a noisy acoustic environment, and understand a talker’s intent and emotional state. Prevailing theories posit that the auditory system relies on the stimulus-driven timing of spikes in the auditory nerve, termed phase locking, to estimate pitch. Recent behavioral results, however, suggest that pitch can still be perceived at high frequencies, where phase-locked information should be highly degraded or nonexistent. To address this discrepancy, we combined behavioral testing methods with computational models of the early auditory system to probe how listeners can achieve accurate pitch discrimination at high frequencies. Optimal decoding of all available auditory-nerve information resulted in a pattern of predictions that matched (but greatly outperformed) human perceptual performance. Understanding how pitch is coded across the frequency range may help in the quest to restore accurate pitch perception in people with impaired hearing and cochlear implants.

## Introduction

Pitch is a primary perceptual dimension of sound. It plays a key role in the perception of music, where it constitutes the basis of melody and harmony [1], as well as in the perception of speech, where it has important suprasegmental functions and conveys information about talker identity [2–4]. Pitch also facilitates auditory scene analysis, helping listeners to segregate simultaneous harmonic sounds [5,6] or to understand speech in complex backgrounds [7]. Although sensitivity to pitch and regular harmonic structure has been demonstrated in auditory cortex of humans [8–10] and other mammals [11,12], theories of the neural basis of pitch perception diverge as early as the auditory nerve. “Place” or “rate-place” theories contend that pitch is derived by analysis of the spatial pattern of average firing rates of auditory-nerve fibers, in which information about the frequency content of a stimulus is encoded via the basilar membrane’s frequency-to-place (or tonotopic) mapping [13,14]. “Temporal” theories suggest instead that pitch is derived from temporal information, including temporal fine structure (TFS) information encoded in inter-spike intervals by the phase-locking properties of auditory-nerve fibers and other temporal information, such as envelope modulation [13,15,16]. “Spatiotemporal” or “spectrotemporal” theories, motivated by the fact that neither place nor temporal theories account well for all pitch phenomena, propose that both the frequency-to-place mapping and TFS information play crucial roles in pitch perception [13,17–20].

The simplest pitch-evoking stimulus is the pure tone, and it is well known that frequency discrimination of pure tones degrades as the stimulus frequency increases beyond 2–3 kHz [21,22]. Because phase locking in the auditory nerve also weakens with increasing stimulus frequency beyond 2–3 kHz [23–26], it has often been argued that frequency discrimination relies on a temporal code. Ideal-observer analysis of simulated auditory-nerve responses suggests that the rolloff of phase locking in the auditory nerve can account well for the dependence of pure-tone frequency discrimination on stimulus frequency in humans [27,28]. However, no direct evidence regarding the lowpass characteristic of phase locking is available in humans, with estimates based on comparative studies, electrophysiology, and psychophysics of the “upper limit” of useful phase locking ranging quite widely from 1.5 kHz up to 8–12 kHz [21,23,25,29]. In addition, new behavioral results [30] have resulted in considerable uncertainty surrounding the extent to which the deterioration of frequency discrimination at high frequencies truly reflects the underlying rolloff of auditory-nerve phase locking to TFS. Nevertheless, at a sufficiently high (although unknown) frequency, no usable phase-locked information should be available in the auditory nerve. At such a point, it is generally believed that a rate-place code for frequency becomes dominant [21].

Harmonic complex tones (HCTs), which are comprised of pure tones whose frequencies are integer multiples of a common fundamental frequency (F0), are a more complicated but more natural pitch-evoking stimulus. Voiced speech and musical instrument sounds are examples of HCTs. Rate-place information is generally thought to be available for lower-ranked harmonics, but not for higher-ranked harmonics, due to the filtering that occurs in the cochlea. The transition between these lower, spectrally resolved, harmonics and the higher, spectrally unresolved, harmonics is also subject to debate but, depending on the definition, is thought to occur somewhere between the 7^th^ and 10^th^ harmonic, at least for F0s of 100 Hz and above [13,31–35]. Behaviorally, F0 discrimination is best when some harmonics lower than the 10^th^ are present [31–34]. For this reason, we concentrate on HCTs that are restricted to a limited number of harmonics in the range from the 6^th^ to 10^th^.

Whereas the link between coding of TFS information in the auditory nerve and frequency discrimination of pure tones is relatively straightforward, the link between coding of temporal information and F0 discrimination of HCTs is considerably more complicated. Here, we use the term “temporal information” to refer both to TFS at the harmonic component frequencies and to other (slower) periodicities, primarily at the F0, evoked by peripheral interactions between components. Discrimination thresholds of such HCTs composed of harmonics from the 6^th^ to 10^th^ are typically poorer by approximately a factor of 5 if the component frequencies are all above ~8 kHz than if they are at lower frequencies [33,36–38]. Qualitatively, this effect is consistent with temporal theories of pitch, which predict that performance should degrade as phase locking to component frequencies (and thus the availability of TFS information about component frequencies) degrades at higher frequencies. Conversely, this effect is qualitatively *inconsistent* with rate-place theories of pitch. In relative terms, cochlear filters remain sharp at high frequencies; thus, the pattern of average auditory-nerve firing rate across the tonotopic axis should be informative about F0 at both low and high frequencies. For spectrotemporal theories of pitch, predictions are less clear because loss of phase locking to TFS may be counterbalanced by relatively sharper auditory filters at high frequencies. Although the difference in performance at low and high frequencies is qualitatively consistent with temporal theories, the magnitude of degradation in F0 discrimination performance at high frequencies (about a factor of 5) is surprisingly small, given that phase-locked responses to TFS above 8 kHz seem unlikely to convey sufficient information to derive accurate estimates of F0 in HCTs [13]. In the absence of TFS information at high frequencies, listeners must instead be relying on temporal-envelope periodicities at the F0, evoked by peripheral interactions between stimulus components, or they must switch to a rate-place code [38,39]. However, it is generally believed that listeners cannot perform pitch discrimination by comparing rates of temporal-envelope cues for high F0s/rates above about 700 Hz [38–42].

If a rate-place code is used at high frequencies, then the presence of spectrally resolved harmonics ought to be a necessary condition for accurate F0 discrimination at high frequencies. Consistent with this prediction, listeners do not achieve accurate F0 discrimination for stimuli at high frequencies when all stimulus harmonics are unresolved [33]. Another way to reduce access to resolved harmonics is to present target HCTs in the context of spectrally overlapping masker HCTs [43–47]. In cases where a sufficient number of harmonics are presented simultaneously in the same frequency region, rate-place cues for resolved harmonics may be reduced or eliminated, insofar as any peaks in an average rate profile would not unambiguously reflect the presence of a single target component, even though the harmonic numbers of the target remain the same. Such stimuli should result in particularly poor F0 discrimination thresholds at high frequencies if listeners are using a rate-place code for F0 discrimination, as compared to F0 discrimination at low frequencies where a temporal code with access to TFS information may be more robust to the presence of HCT maskers.

In the present study, we tested the hypothesis that high-frequency F0 coding is based on a rate-place code using a mixture of behavioral and computational modeling methods. First, we present simulations of auditory-nerve responses to HCTs at low and high frequencies to develop a better understanding of the types of temporal and rate-place cues available for F0 discrimination at low and high frequencies. Next, we present behavioral data that were collected to test the hypothesis that mixtures of HCTs should result in particularly poor F0 discrimination at high frequencies–a pattern of results that would be consistent with the use of a rate-place code at high frequencies. Finally, we combine the auditory-nerve models with ideal-observer analysis to generate optimal frequency difference limens (FDLs) and F0 difference limens (F0DLs) for isolated pure and complex tones, over a wide range of frequencies, and compare these predictions to behavioral thresholds from the literature.

## Results

### Peripheral representation of HCTs at high frequencies

Although many studies have recorded auditory-nerve responses to the types of HCT stimuli used in pitch experiments [13,20,48,49], these have not included HCTs at the very high frequencies used in recent human psychophysical work [36–38]. Moreover, recent work has revealed significant differences in peripheral coding between humans and the smaller mammals commonly used in auditory physiology experiments [29,50,51], raising questions as to how useful auditory-nerve recordings of pitch-evoking stimuli in animals such as guinea pigs or chinchillas are in understanding how pitch stimuli are represented in the human auditory periphery. To develop a better understanding of the availability and quality of different F0-related cues at high frequencies as compared to low frequencies in the human auditory periphery, we simulated human auditory-nerve responses to HCTs at low and high frequencies using a modern phenomenological model of the auditory nerve [52], with parameters adjusted to match what is known about human cochlear tuning. This was done after first validating that the cat version of the model could qualitatively replicate key data from relevant studies in cat (S1 Text).

**Fig 1:**
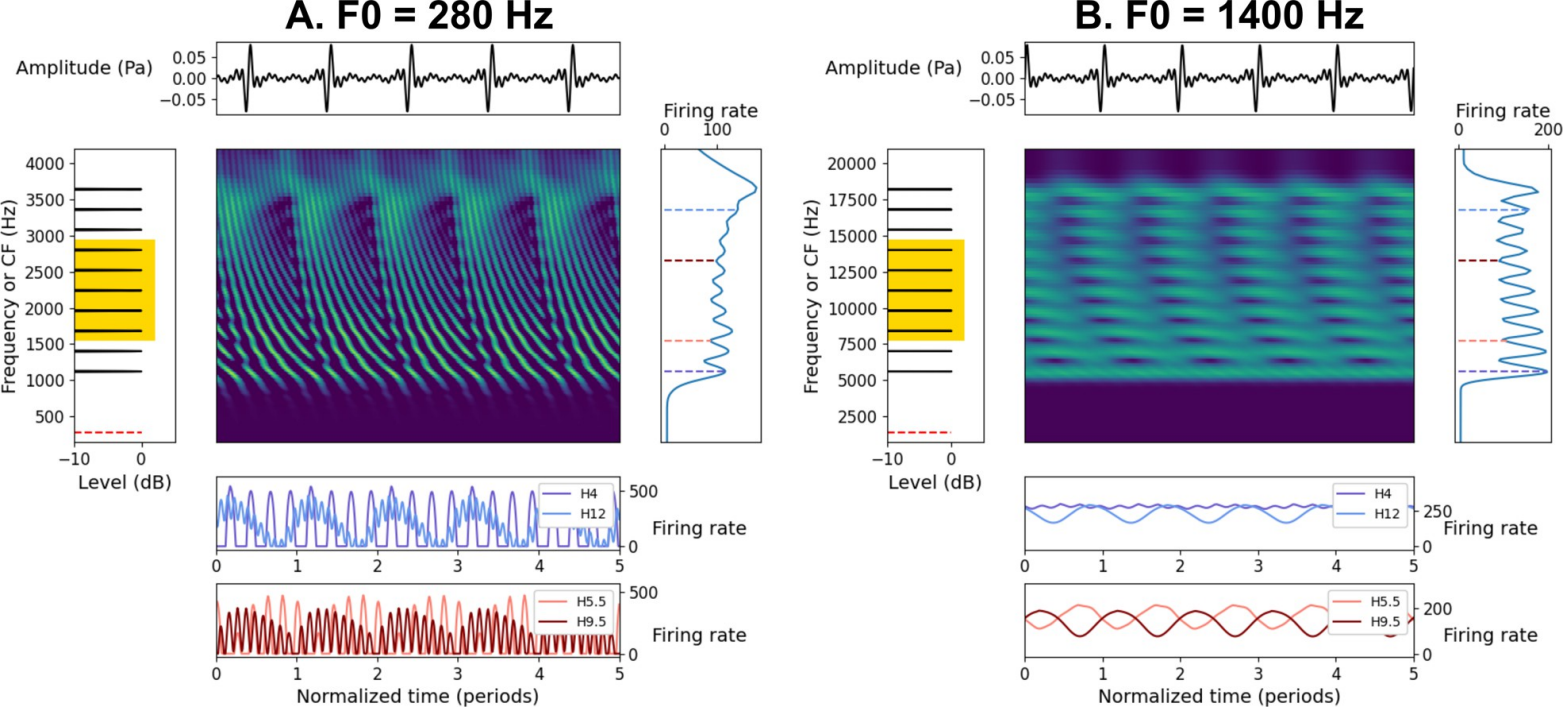
Representation of complex tones in a simulated auditory nerve. (A). Simulated responses of a population of high-spontaneous-rate auditory-nerve fibers [52] for five periods of a sine-phase HCT composed of harmonics 4–13 at a level of 50 dB SPL per component and an F0 of 280 Hz. The middle panel shows a “neurogram”, or a plot of instantaneous firing rate as a function of time and characteristic frequency. In the neurogram, color (from purple [low] to yellow [high]) indicates the instantaneous firing rate analogous to color indicating intensity in a spectrogram. The top and left panels show the temporal waveform and spectrum, respectively, of the acoustic stimulus. In the left panel, the yellow box highlights the frequency ranges used in the behavioral experiments. The bottom two panels and right panels show the responses of individual nerve fibers over time and the response profile averaged over time, respectively, of the simulated neural response. The bottom two panels show responses for auditory-nerve fibers tuned to component frequencies (4F0, purple; 12F0, blue) or tuned between component frequencies (5.5F0, pink; 9.5F0, maroon). (B). Same as A, except for an F0 of 1400 Hz.

Low-numbered harmonics were at least partially resolved by the model filterbank (emulating the mechanical filtering of the basilar membrane) at both low and high F0s, as reflected by prominent peaks in the pattern of average firing rates over characteristic frequency (CF) in the auditory-nerve population (Fig 1A and 1B, right panel). At low F0s, model fibers tuned to resolved components also demonstrated robust phase locking to the underlying TFS, whereas fibers tuned to unresolved components instead responded with a prominent modulation at F0 (Fig 1A, bottom panel). At high F0s, component frequencies were too high to produce phase-locked responses in the model auditory nerve. As a result, model fibers tuned to resolved components showed responses with little in the way of temporal structure, whereas fibers tuned to unresolved harmonics showed strong modulations at F0 (Fig 1B, bottom panel). At both low and high frequencies, model fibers tuned between resolved components showed responses modulated at F0 (Fig 1A and 1B, bottom panel).

The model simulations suggest that if temporal information is utilized at high frequencies, then it is likely to be based on the envelope modulations at F0, rather than on the phase-locked responses to individual harmonics (an observation further reinforced by our ideal-observer modeling below). However, several lines of evidence suggest that humans cannot utilize that envelope information effectively. First, studies using HCTs composed of unresolved harmonics (i.e., all harmonics above the 10^th^) or modulated noises show that the pitch of such stimuli, which is conveyed exclusively by temporal-envelope cues, is weak, yields poor F0 discrimination, and is non-existent for F0s above about 700 or 800 Hz [32–34,40,42]. Second, melody discrimination is possible at very high frequencies (> 7.5 kHz) for HCTs with F0s between 1 and 2 kHz, but when the harmonics are shifted to produce inharmonic tones, performance drops to near chance, even though the temporal-envelope cues are maintained [39]. Third, both melody perception and F0 discrimination remain accurate at high frequencies when odd and even harmonics are presented to opposite ears, even though the temporal-envelope repetition rate in each ear is thereby doubled [38,39]. These lines of evidence, which we evaluate and reconsider further in light of our own results below, suggest that at least some information in the temporal response pattern of the auditory nerve at high frequencies may not be utilized perceptually.

### F0 discrimination is affected by HCT maskers at low and high frequencies

If listeners are not using temporal information to perform F0 discrimination at high frequencies, then they must be relying on rate-place information, which in turn relies on the presence of some spectrally resolved harmonics. We tested this idea by attempting to restrict the availability of resolved harmonics via the addition of concurrent HCT maskers. We hypothesized that this stimulus manipulation would yield particularly poor performance at high frequencies (where resolved harmonics are necessary due to the use of a rate-place code) as compared to low frequencies (where resolved harmonics may not be necessary, due to the additional availability of TFS cues, even in the presence of the masker).

#### Experiment 1

We began by measuring F0 discrimination at both low and high frequencies and by determining how a single HCT masker impaired F0 discrimination. Experiment 1 measured F0 discrimination thresholds for bandpass-filtered HCTs at low frequencies (F0 = 280 Hz, frequency range = ~1.5–3.0 kHz) and high frequencies (F0 = 1400 Hz, frequency range = ~8–14 kHz). Two conditions were tested: ISO, in which test tones (target and reference) were presented in isolation (except for masking noise in the background), and GEOM, in which target tones with masking noise were presented concurrently with a spectrally overlapping HCT masker with an F0 that was geometrically centered between the F0s of the reference (lower-F0) and the target (higher-F0) tone. The masking noise was broadband threshold-equalizing noise (TEN) [53] with a level within the estimated equivalent rectangular bandwidth (ERB) of the human auditory filter around 1 kHz [54] that was 10 dB below the level per component of the HCTs. Two stimulus variants were tested. In the first variant (Experiment 1a), the test tones contained only harmonics 6–10 of the F0. To help rule out the possibility that listeners were using the spectral edge of the stimulus, rather than the F0, to complete the task, a second variant (Experiment 1b) was tested, in which the test tones contained all harmonics of the F0 up to the Nyquist frequency (i.e., half the sampling rate), but were bandpass filtered with a zero-phase 12^th^ order Butterworth bandpass filter passing harmonics 6–10 of the nominal F0.

**Fig 2:**
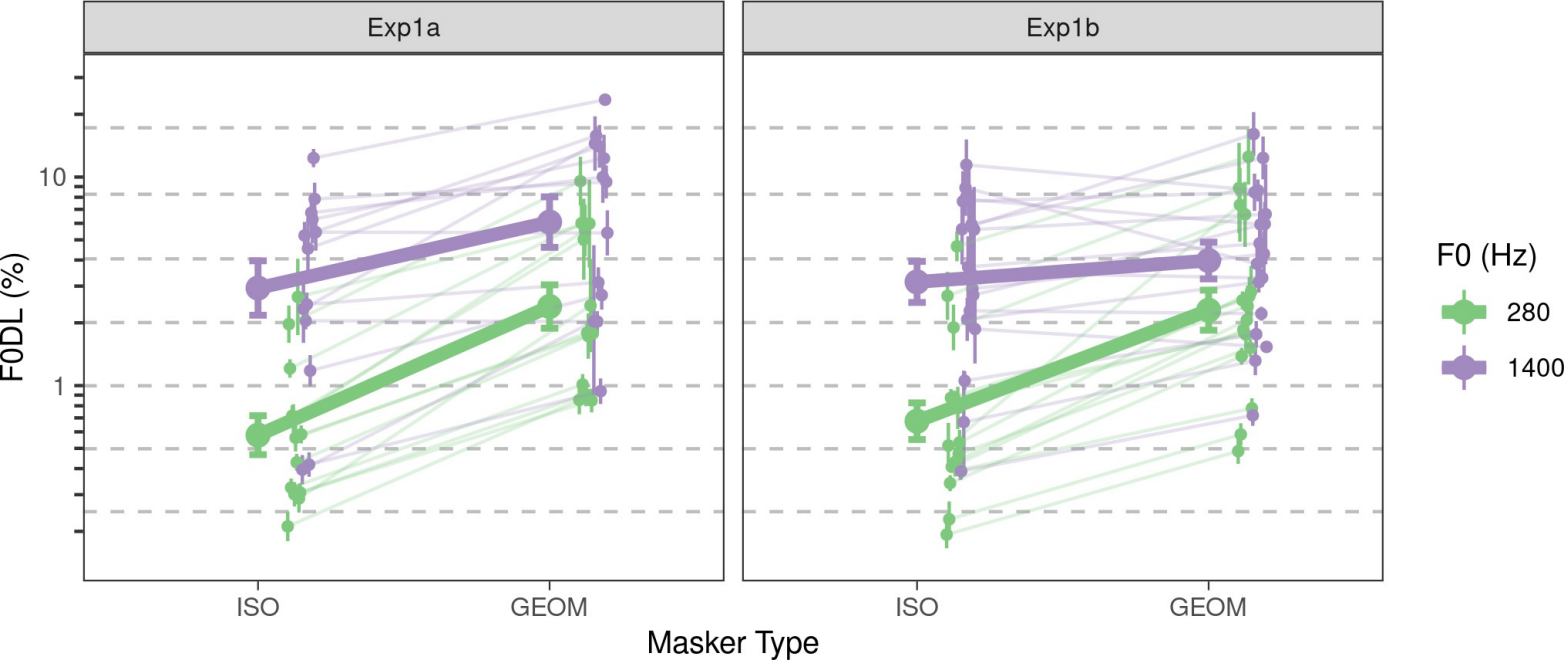
Behavioral results from Experiment 1. The left panel (Exp1a) shows results with only harmonics 6–10 present in the target; the right panel (Exp1b) shows results with the bandpass filtered target, with harmonics 6–10 in the passband. Large filled circles and error bars indicate the average F0DL and ±1 standard error of the mean (SEM). The small filled circles and error bars indicate individual F0DLs and ±1 SEM for each participant.

The results of Experiment 1 are plotted in Fig 2. An analysis of variance (ANOVA) revealed significant main effects of F0 (low or high) [*F*(1, 22.98) = 54.57, *p*<0.001] and masker (present or absent) [*F*(1, 22.46) = 149.37, *p*<0.001] as well as significant two-way interactions between F0 and masker [*F*(1, 22.29) = 44.71, *p*<0.001] and between masker and experiment (Experiment 1a or Experiment 1b) [*F*(1, 130.42) = 9.02, *p* = 0.013]. No other model terms reached significance. The significant main effects reflected the trends observed in Fig 2 that listeners achieved better (lower) F0 discrimination thresholds at low frequencies than at high frequencies both for the ISO condition [estimated ratio = 5.14, *F*(1, 22.82) = 63.41, *p*<0.001] and the GEOM condition [estimated ratio = 2.14, *F*(1,22.82) = 29.80, *p*<0.001] and that they achieved better F0 discrimination thresholds in the absence (ISO) than in the presence (GEOM) of the masker for both low frequencies [estimated ratio = 3.70, *F*(1, 22.69) = 210.15, *p*<0.001] and for high frequencies [estimated ratio = 1.54, *F*(1, 22.49) = 17.00, *p*<0.001].

Our initial hypothesis was that the addition of a masker (i.e., the GEOM condition) would reduce the availability of rate-place cues and so would result in particularly poor F0 discrimination at high frequencies. In fact, contrary to our hypothesis, an interaction contrast test revealed that the difference in performance between the ISO and GEOM conditions was larger at *low* frequencies than at high frequencies [estimated ratio of ratios = 2.40, *F*(1, 22.29) = 42.71, *p*<0.001]. There are many possible explanations as to why the GEOM masker worsened performance more at low frequencies than at high frequencies. One possibility is that the smaller effect of the GEOM masker at high frequencies reflects a ceiling effect for pitch discrimination in our task. That is, for large F0 differences, listeners may have relied on changes in gross spectral cues instead of on TFS information or spectral details. As a result, there may have been an upper limit to how poor F0DLs could be in our stimulus conditions, and if listeners reached such a limit in the high-frequency GEOM condition, the ISO-GEOM ratio in the high-frequency condition may have underestimated the true impact of the GEOM masker at high frequencies. Another possibility is that the GEOM masker did not achieve its intended goal of eliminating representations of resolved harmonics in the neural response to the stimulus; this possibility is explored further via modeling described below.

Somewhat different stimuli were used in Experiment 1a and Experiment 1b. Specifically, the strong spectral edges cues present in the stimuli for Experiment 1a (which were systematically related to the F0 on each trial) were replaced with sloping spectral edges in Experiment 1b (which were *not* systematically related to the F0 on each trial) by using a bandpass filter. As indicated by the significant interaction between masker and experiment, this difference affected performance, with the effect of the masker being approximately 1.4 times larger on average in Experiment 1a than in Experiment 1b [estimated ratio of ratios = 1.4, *F*(1, 130.42) = 9.02, *p* = 0.016]. From visual inspection of Fig 2, the difference between Experiments 1a and 1b appears constrained to the high-frequency GEOM condition. However, the three-way interaction between F0, masker, and experiment was not significant, and a series of pairwise contrasts between Experiments 1a and 1b for each of the conditions did not reveal any significant differences after correction for multiple comparisons (all *p*>0.054). Collectively, the small size of the observed differences between Experiment 1a and Experiment 1b, and the fact that performance was, if anything, better in Experiment 1b than in Experiment 1a, suggests that listeners were not using spectral edge cues in Experiment 1a.

### Experiment 2

It is possible that the GEOM masker may not have achieved its intended goal of eliminating rate-place representations of resolved target harmonics in the neural response. Experiment 2 attempted to address this possibility by measuring the target-to-masker ratio (TMR) that listeners required to discriminate the F0 of HCTs presented concurrently with *two* spectrally overlapping HCT maskers. The test tones (reference and target) had F0s that were separated by 1.5 or 2.5 times the F0DL measured for each participant individually without a masker. The masker tones had F0s that were below and above the F0 of the test tones (by between 5.25–7.25 semitones, selected randomly on each trial with a uniform distribution), and auditory-nerve simulations (see below) confirmed that the target harmonics were unlikely to be spectrally resolved at a TMR of 0 dB (equal-amplitude target and masker components). The targets and maskers were both synthesized in the same way as the tones in Experiment 1b (i.e., containing all harmonics of their F0 but bandpass filtered to attenuate all but harmonics 6–10 of the target or 5–11 of the maskers).

**Fig 3:**
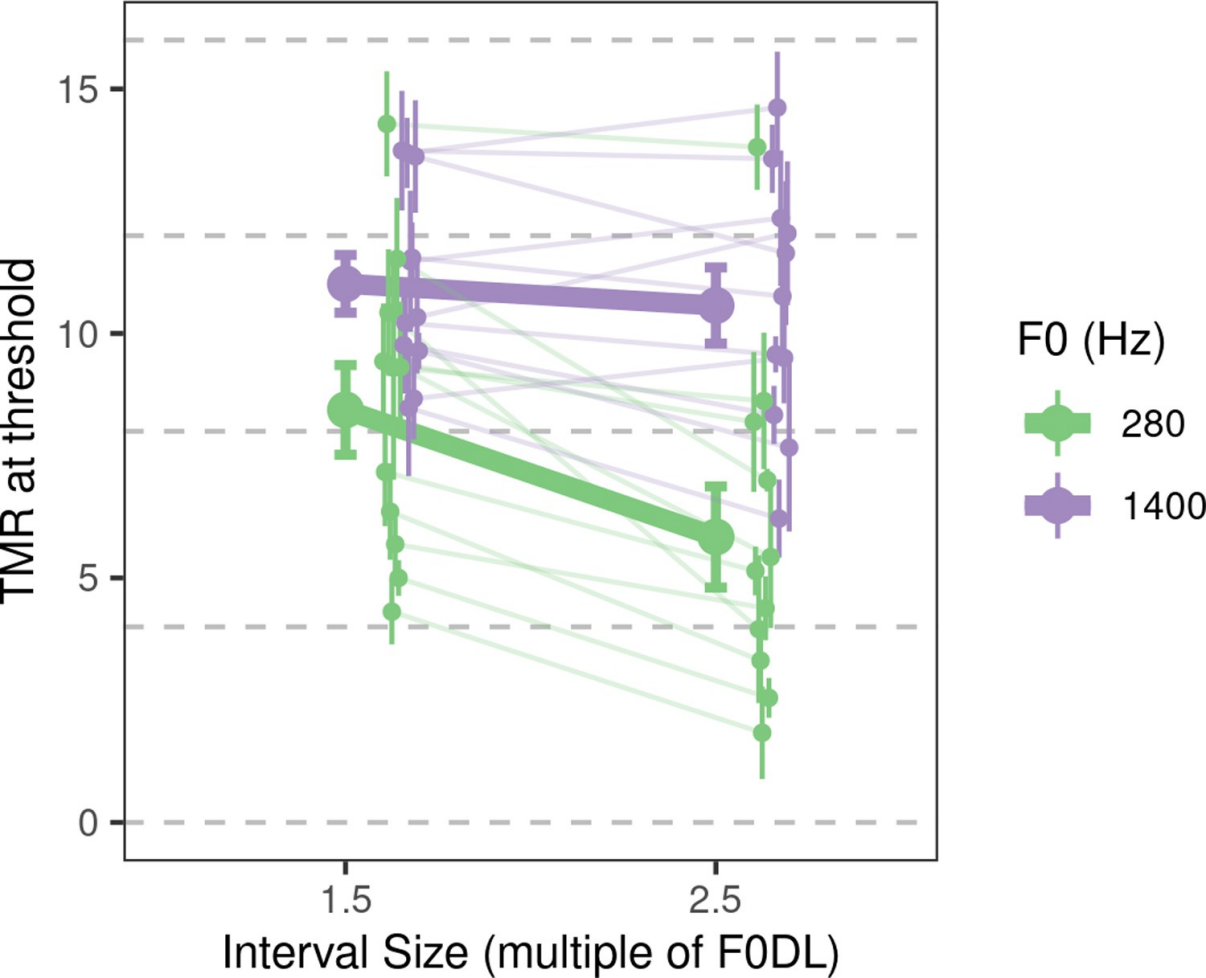
Behavioral results from Experiment 2. Results from Experiment 2. Large filled circles and error bars indicate the average TMR and ±1 standard error of the mean (SEM). The small filled circles and error bars indicate individual F0DLs and ±1 SEM for each participant.

The results of Experiment 2 are shown in Fig 3. An ANOVA revealed significant main effects of F0 (low or high) [*F*(1, 10.00) = 11.78, *p* = 0.013] and interval size (1.5 or 2.5 F0DL) [*F*(1, 10.00) = 17.43, *p* = 0.0057] as well as a significant interaction between F0 and interval size [*F*(1, 10.00) = 6.77, *p* = 0.026]. Listeners achieved considerably lower TMRs in low-frequency conditions than in high-frequency conditions [estimated difference = −3.66 dB, *F*(1, 10.00) = 11.78, *p* = 0.019]. This frequency effect was present even though the difference in F0 was set for each listener based on their own F0DLs from the corresponding ISO condition in Experiment 1, thus nominally equating difficulty across low- and high-frequency conditions in the absence of the masker. In other words, the presence of two HCT maskers interfered more with pitch discrimination at high frequencies than at low frequencies. Under the assumption that rate-place cues for F0 were successfully eliminated by the DBL masker, this finding is qualitatively consistent with our hypothesis that F0 discrimination at high frequencies is based on a rate-place code and so should be more strongly disrupted by the reduction or elimination of spectrally resolved harmonics. This conclusion, and the assumptions underlying it, are considered in more detail in the following section.

As expected, listeners generally performed better with the larger interval size (2.5 F0DL) than with the smaller interval size (1.5 F0DL), as confirmed by a contrast test [estimated difference = −1.52 dB, *F*(1, 17.43) = 17.43, *p* = 0.0076]. However, an unexpected interaction between F0 and interval size revealed that the larger interval size yielded better performance at low frequencies [estimated difference = −2.60 dB, *F*(1, 1.00) = 21.21, *p* = 0.0049] but not at high frequencies [estimated difference = −0.44 dB, *F*(1, 10.00) = 0.68, *p* = 0.43]. An interaction contrast test comparing the size of the interval effect at low and high frequencies was, after correction, marginally significant [estimated difference of differences = 2.16 dB, *F*(1, 10.00) = 6.77, *p* = 0.053], providing modest evidence that the size of the interval effect differed between low and high frequencies, although in both cases the trend was in the same direction, with a larger interval producing a lower TMR at threshold.

### Auditory-nerve modeling highlights the complexities of HCT mixtures

In the development and interpretation of Experiments 1 and 2, we assumed that the addition of concurrent HCT maskers would reduce or eliminate rate-place cues for target components in the auditory-nerve response. Under this assumption, we expected that the HCT maskers would impact performance more at high frequencies (where we hypothesized that rate-place cues are necessary for good performance) than at low frequencies (where TFS information might still convey useful information even in the presence of the maskers). Instead, we found that the maskers had a larger negative effect at low frequencies in Experiment 1 and a larger negative effect at high frequencies in Experiment 2. Our observed pattern of data can be qualitatively explained by assuming that (1) rate-place cues were not eliminated by the GEOM masker in Experiment 1 (resulting in better-than-expected performance at high frequencies), (2) rate-place cues *were* eliminated (or heavily degraded) by the DBL masker in Experiment 2 (resulting in poorer than expected performance at high frequencies), and (3) temporal cues were relatively less affected by the maskers than were rate-place cues (explaining why the DBL masker had less effect at low than at high frequencies). To explore the validity of these assumptions, we simulated auditory-nerve responses to our HCT mixture stimuli from Experiments 1 and 2 and visualized the availability of different cue types for each masker type and F0.

**Fig 4:**
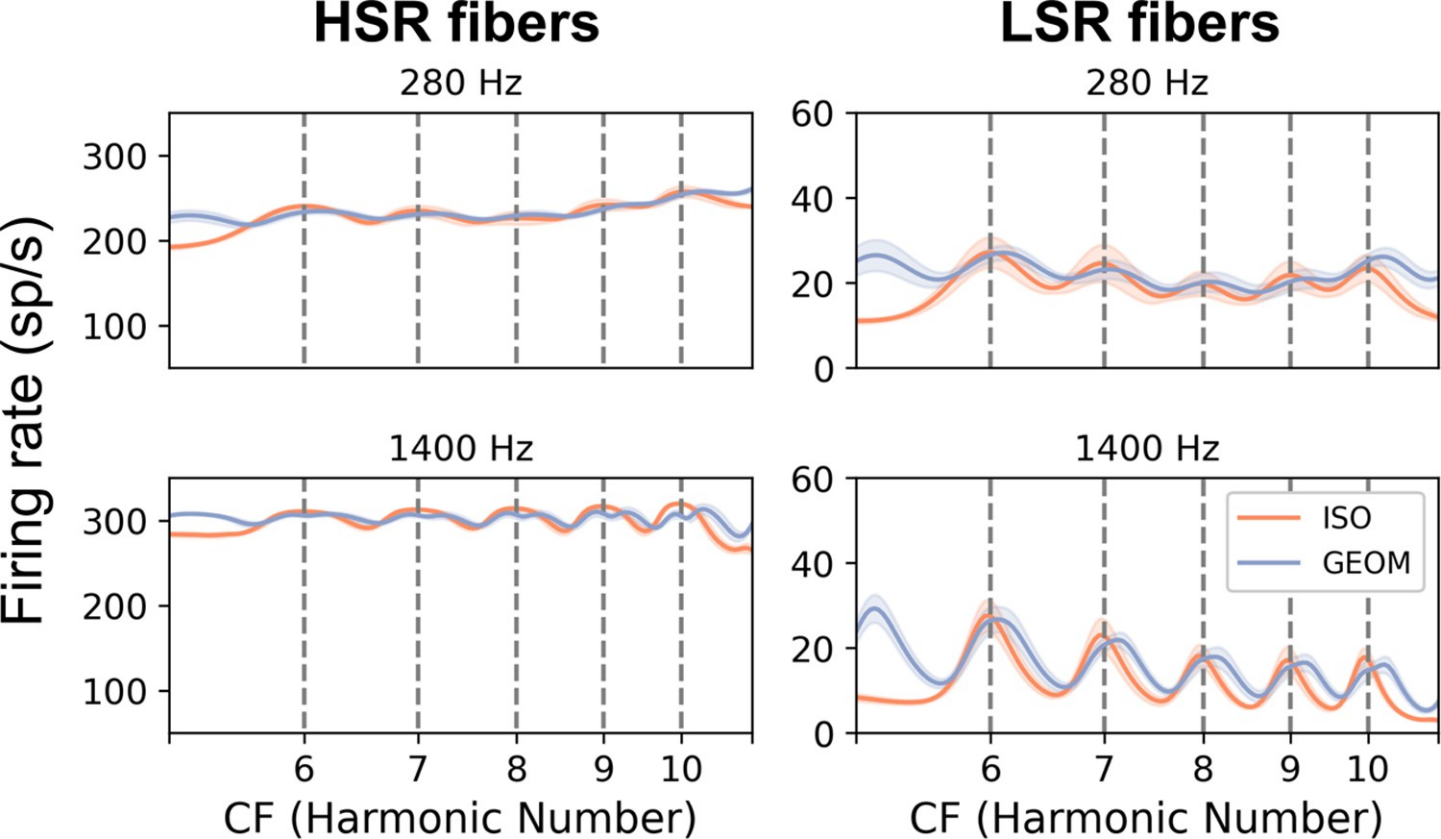
Comparison of the availability of spectrally resolved harmonics at low and high frequencies in an excitation pattern simulation. Excitation patterns (average firing rate versus CF) for high-spontaneous-rate (HSR) auditory-nerve fibers (left) and low-spontaneous-rate (LSR) auditory-nerve fibers (right) responding to the stimuli in Experiment 1b based on a computational model of the auditory periphery [52]. The solid curve indicates the average excitation pattern while the filled area around the curve indicates ±1 standard deviation (over 10 simulations with different samples of masking noise and level roving). Vertical dashed lines indicate the frequencies of target harmonics. The F0 difference between the target and masker was 3%. See Materials and Methods for more details on the simulations.

For Experiment 1, we simulated responses to the targets in alone and in the presence of the GEOM masker for populations of high-spontaneous-rate and low-spontaneous-rate auditory-nerve fibers and visualized the average-rate responses (i.e., excitation patterns). As can be seen in Fig 4, for high-spontaneous rate fibers, resolved harmonics were only barely visible in the average-rate response for ISO stimuli at both low and high frequencies, as the sound levels were high enough to saturate most simulated high-spontaneous-rate fibers). However, for low-spontaneous-rate fibers, resolved harmonics were more clearly visible in the average-rate response to the ISO stimulus at both low and high frequencies, with responses at high frequencies in particular showing strong peaks and valleys corresponding to the frequencies of the target harmonics (this difference at low and high frequencies is consistent with sharper relative tuning in the model at high frequencies, in line with estimates from humans and other species; [50,51]).

At least for the low-spontaneous-rate fibers, the GEOM masker seems to have elicited broad peaks reflecting a combination of target and masker components. These combined peaks evoked by pairs of target and masker harmonics may still be useful for coding F0, insofar as they are still systematically related to the target component frequencies. F0 discrimination based on identifying peaks in excitation patterns might worsen only to a small extent when using these blurred peaks instead of using sharper peaks associated with target components alone [45]. Regardless, the central insight from Fig 4 is that the GEOM masker likely did not achieve its intended goal of eliminating rate-place cues at high frequencies, making interpretation of the resulting behavioral results challenging. Future psychophysical studies could attempt to overcome these limitations in part by exploring other types of maskers, such as (possibly inharmonic) complex tones or filtered noise stimuli tailored to “fill in” the gaps between resolved target components in each interval. Such maskers could also potentially overcome other limitations of the present masker stimuli, such as the potential for strong beats between the harmonics of the target and the harmonics of the maskers (which were often very close in frequency for listeners that achieved good thresholds).

**Fig 5:**
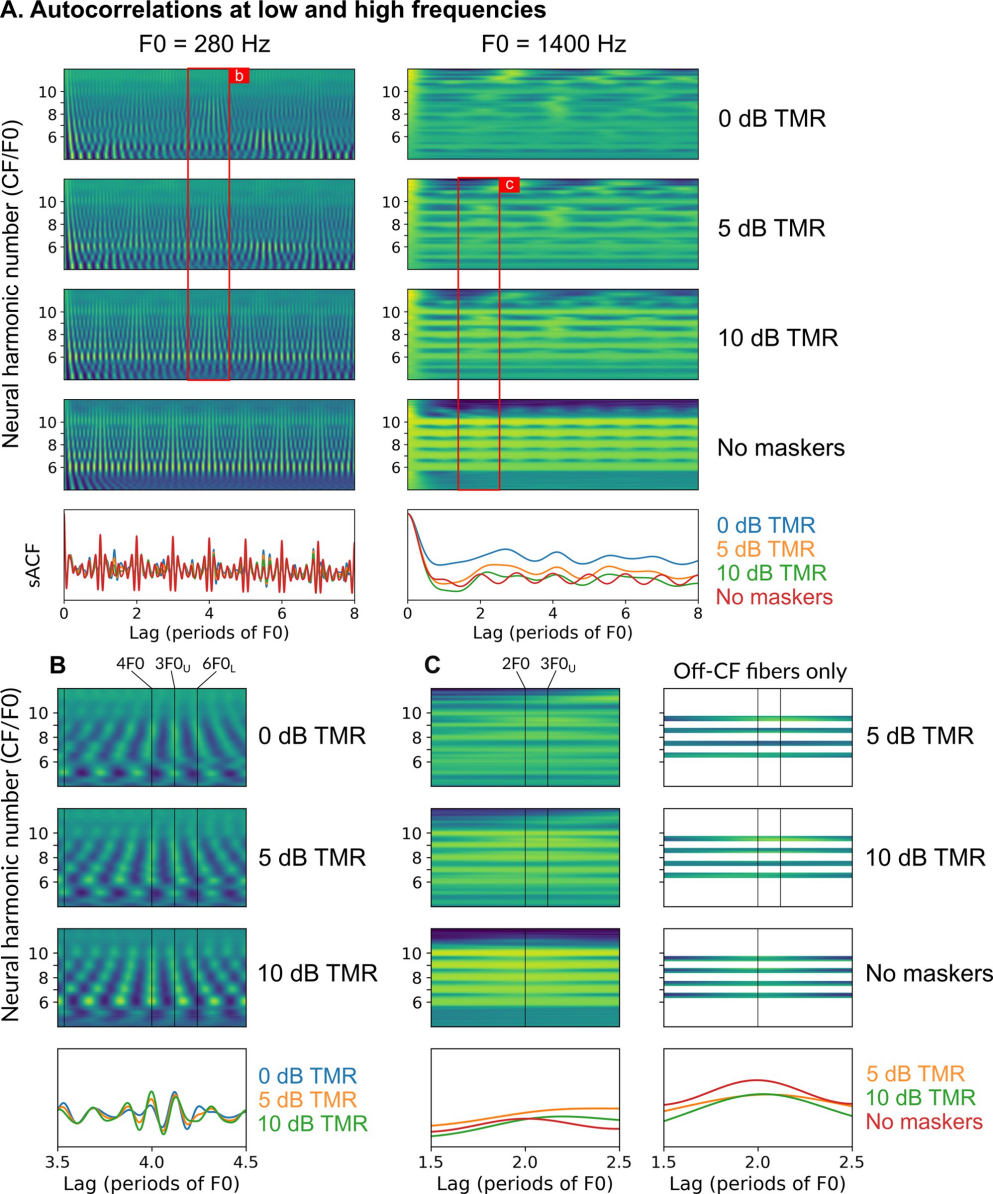
Comparison of simulated autocorrelograms at low and high frequencies for the DBL stimulus. (A) Autocorrelograms for simulated high-spontaneous-rate auditory-nerve-fiber responses [52] for the low-frequency DBL stimulus (left column) and the high-frequency DBL stimulus (right column). Here, one masker F0 was set to 5.5 ST below the target F0 while the other masker F0 was set to 6 ST above the target F0. The figure was constructed by fixing the target F0 and then varying the simulated CF (y-axis) linearly over a fixed range. For each CF, the instantaneous firing rate of the auditory-nerve model was computed and then the autocorrelation of that response was computed. Color (from purple/blue [low] to yellow/green [high]) indicates the autocorrelation value at each lag-CF point, analogous to color indicating intensity in a spectrogram. In each column, the first four panels show autocorrelograms for varying TMRs and the bottom panel shows the summary autocorrelation function (sACF; the sum of the autocorrelograms along the CF axis). Red boxes and labels indicate the zoomed-in views plotted in the following subfigures. (B) A zoomed-in view of autocorrelograms and the sACF for 0, 5, and 10 dB TMR at low frequencies for lags of 3.5 to 4.5 times the period of the target F0. Vertical lines indicate lags corresponding to the periods of each tone in the stimulus or to multiples of those periods (marked using the shorthand “2F0” to refer to the lag twice as long as the period of the tone). Lags corresponding to multiples of the target period are indicated with “XF0”, while lags corresponding to multiples of the masker periods are indicated with “XF0_L_” and “XF0_U_” for the lower and upper maskers, respectively. (c) A zoomed-in view of autocorrelograms and the sACF for 5 and 10 dB TMR and without maskers at high frequencies for lags of 1.5 to 2.5 times the period of the target F0. Vertical lines indicate the same as in the previous subfigure. Only vertical lines corresponding to the target period are plotted in the last subpanel because this simulation was conducted without the maskers. See Materials and Methods for more details on the simulations.

**Fig 6:**
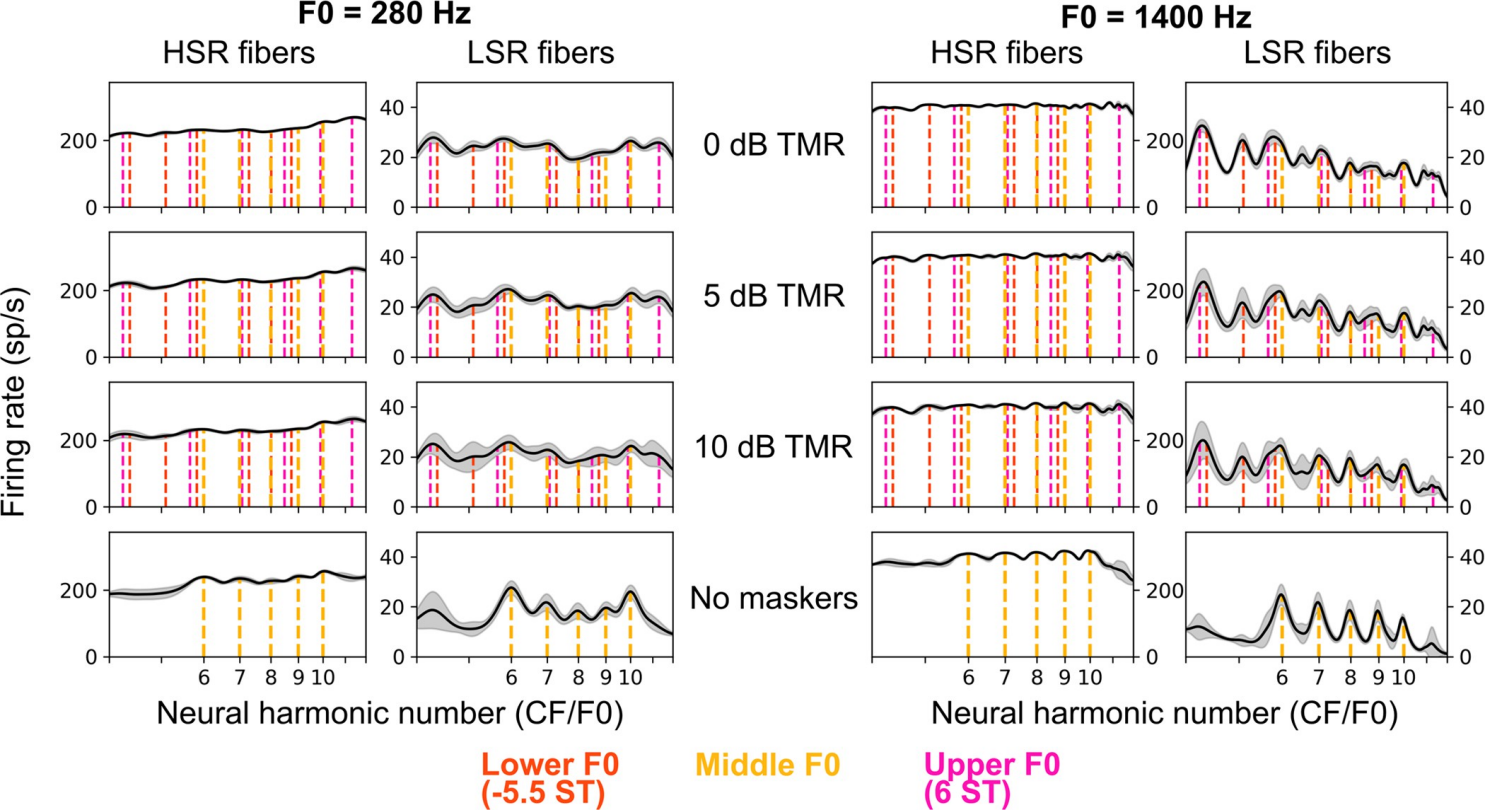
Comparison of excitation patterns at low and high frequencies for the DBL stimulus. Excitation patterns (average firing rate versus CF) for simulated high-spontaneous-rate and low-spontaneous-rate auditory-nerve fibers [52] responding to the low-frequency DBL stimulus (left column) and the high-frequency DBL stimulus (right column) in Experiment 2. The solid curve indicates the average excitation pattern while the filled area around the curve indicates ±1 standard deviation (over 10 simulations with different samples of masking noise and level roving). Vertical dashed lines indicate the frequencies of target components and masker components. Color of the lines indicates which F0 they corresponding to (orange for the lower masker F0, gold for the target F0, and pink for the upper masker F0). See Materials and Methods for more details on the simulations.

For Experiment 2, the key uncertainty in interpreting the results is whether the DBL masker achieved its intended goal of eliminating rate-place cues for resolved target components, and to what extent the masker impacted temporal cues at low frequencies. To probe these questions, we simulated auditory-nerve responses to the DBL stimulus as a function of TMR and visualized the availability of temporal and rate-place cues. Fig 5 shows autocorrelograms (i.e., autocorrelations of auditory-nerve responses over a range of CFs; [48]) of auditory-nerve responses (temporal coding) at both low and high frequencies while Fig 6 shows excitation patterns (rate-place coding) at both low and high frequencies for the DBL stimulus.

In Fig 5, strong evidence for the target F0 consists of vertical ridges at lags corresponding to the F0 (integers on the x-axis) spanning along the range of CFs (y-axis). As can be seen, temporal coding of the target F0 was weak at 0 dB TMR at both low and high frequencies. At low frequencies and at 0 dB TMR, vertical ridges in the autocorrelogram often reflected synchronization to masker components instead of target components (Fig 5B). At more favorable TMRs, vertical ridges corresponding clearly to the target period emerged and the most prominent peaks in the summary autocorrelation (sACF) were located at integer multiples of the target period. By 10 dB TMR, the low-frequency autocorrelogram closely resembled the autocorrelogram for the stimulus without maskers. Coding of the target F0 was much weaker at high frequencies, consistent with the loss of phase locking to the TFS of individual components at high frequencies. Even at 10 dB TMR, temporal coding of the target F0 was considerably less salient than in the absence of the maskers. Fibers tuned near stimulus components conveyed little temporal information about F0 (because they are saturated and do not phase lock to the high-frequency TFS; Fig 1). Fibers tuned between stimulus components still conveyed some temporal information (because they respond to modulations at F0 elicited by beating between adjacent target components in the stimulus; Fig 1), but the resulting autocorrelation peaks were broad and shallow (Fig 5C).

In Fig 6, strong evidence for the target F0 would consist of prominent peaks at each target harmonic (integers on the x-axis). High-spontaneous-rate fibers showed few prominent peaks at any TMRs at either low or high frequencies because the sound levels used in the simulations saturated the high-spontaneous-rate fibers. In contrast, low-spontaneous-rate fibers showed many prominent peaks in the excitation patterns. At 0 dB TMR, these peaks corresponded to masker components or mixtures of target and masker components. At more favorable TMRs, these peaks more clearly reflected target frequencies. However, even with masker components attenuated by 10 dB (i.e., 10 dB TMR), the peaks elicited by target components still had smaller peak-to-valley ratios than corresponding peaks in the excitation pattern without maskers (bottom row), masker components outside the stimulus passband still elicited strong peaks, and peaks near masker components within the stimulus passband still influenced the shape of peaks near target components. This simulation demonstrates that the notion of “resolved harmonics” in the context of mixtures of HCTs, where auditory neurons are responding to mixtures of components in complicated ways, is problematic and lacks a clear definition [44].

Figs 5 and 6 emphasize the complexities inherent in interpreting the results of Experiment 2. Both temporal and rate-place cues were likely strongly impacted by the presence of the DBL masker. If we assume that the *same* mechanism is used at low and high frequencies to decode the target F0, then predictions are relatively clear. If a temporal code is used at low and high frequencies, thresholds with the DBL masker ought to be better at low frequencies than at high frequencies, because temporal cues at low frequencies are much more robust to the presence of the DBL masker than are temporal cues at high frequencies (Fig 5). If instead a rate-place code is used at low and high frequencies, thresholds with the DBL masker ought to be better at high frequencies than at low frequencies, because sharper relative tuning at high frequencies leads to clearer rate-place cues for F0 at high frequencies than at low frequencies (Fig 6). In our behavioral data, we observed that performance was better at low frequencies than at high frequencies with the DBL masker (Fig 3), a pattern of results that is nominally consistent with the temporal-code prediction. However, if different mechanisms are used at low and high frequencies to decode target F0, then predictions are less clear because comparing the relative fidelity of temporal and rate-place cues is non-trivial.

### Predicting performance with an ideal-observer model

The behavioral experiments provide novel data on how concurrent and spectrally overlapping HCT maskers affect pitch discrimination at both low and high frequencies. However, the results are equivocal in their support of the hypothesis that pitch discrimination is based on a rate-place code at high frequencies. In Experiment 1, adding a single HCT masker impaired pitch discrimination more at low frequencies than at high frequencies (contrary to our hypothesis), whereas in Experiment 2, adding a double HCT masker impaired pitch discrimination more at high frequencies than at low frequencies (consistent with our hypothesis). Moreover, the simulations illustrated in Figs 4–6 emphasize that adding HCT maskers to an HCT target is likely to affect both temporal and rate-place cues for F0 in complicated ways, making it difficult to interpret the effect of masker in terms of either set of cues.

An alternative approach to probing the neural underpinnings of high-frequency F0 discrimination is to focus on the difference in performance at low and high frequencies for isolated HCTs (which evoke less complicated responses than HCT mixtures) and attempt to determine whether the magnitude of that difference is consistent with the predictions of either temporal or rate-place models of pitch. To this end, we employed three existing computational models of the human auditory periphery [27,52,55] to predict FDLs for pure tones and F0DLs for the isolated HCTs from Experiment 1a using ideal-observer analysis. Simulations of pure-tone FDLs were included to provide direct comparisons with prior work using similar methods [27,28,56]. Thresholds were based on simulated responses from high-spontaneous-rate fibers at 40 CFs spanning a 1.5-octave range around the target frequency (FDLs) or 40 CFs spanning a ~1.1-octave range around the stimulus passband (F0DLs; see Materials and Methods for details). Thresholds were generated for two observer types: an all-information observer and a rate-place observer. Whereas the all-information observer utilized all information in the simulated auditory-nerve response optimally, the rate-place observer averaged the simulated auditory-nerve response over time and then utilized that time-averaged information optimally (see Materials and Methods for details). These two observers allowed us to establish an optimal bound on performance in the modeled tasks (all-information observer) and to determine how much predicted performance was affected by discarding all temporal information from the simulated auditory-nerve responses (rate-place observer).

#### Frequency discrimination

**Fig 7:**
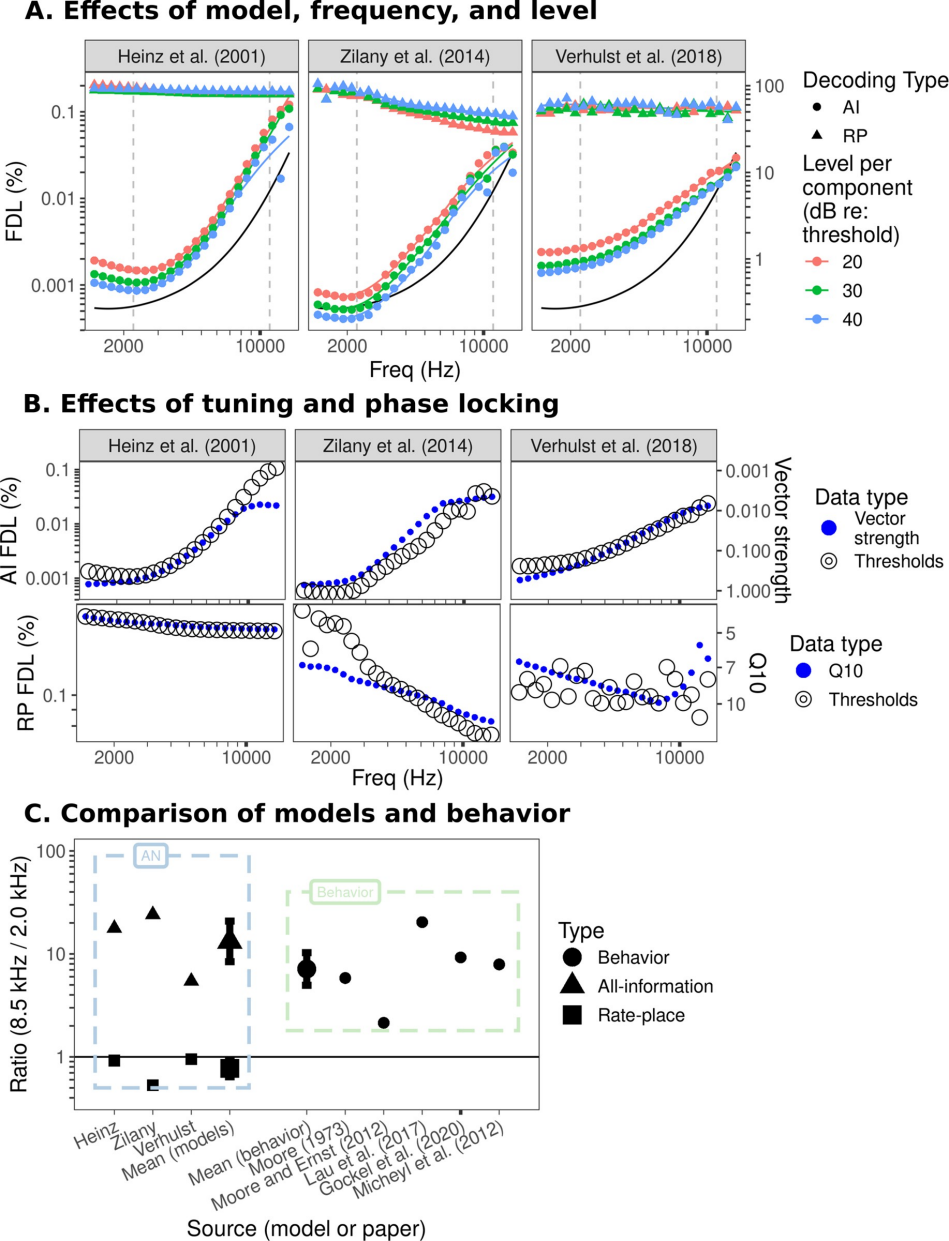
Ideal-observer predictions for pure-tone frequency discrimination. Results of the frequency discrimination simulations. (A) Simulated FDLs versus frequency for a pure tone in each auditory-nerve model. Simulations in this panel include no parameter roving. Points indicate the simulated FDLs at a particular frequency while lines indicate a locally estimated scatterplot smoothing (LOESS) fit to the simulated FDLs. The solid black line indicates the predicted FDLs from Micheyl et al. [58] scaled by a factor of 0.002 (to roughly match the low-frequency side of the curve to the best-performing model predictions from the present study). The axis on the right-hand side corresponds to the unscaled FDLs predictions from Micheyl et al. [58]. (B) Simulated all-information FDLs and vector strength (top row) and simulated rate-place FDLs and Q10 (bottom row) versus frequency with a double y-axis. To choose the warping on the y-axis for vector strength and Q10, linear models were fit to predict log-transformed FDLs as a function of log-transformed reciprocals of vector strength (Q10) for the all-information FDLs (rate-place FDLs). The fitted regression equations were then used to warp the y-axes. In other words, we warped the y-axes for vector strength and Q10 to maximize overlap with the FDL predictions (across all three models) in order to visually demonstrate the relationship between vector strength and Q10 and the simulated FDLs. (C) Ratio of simulated FDLs at 8.5 kHz and 2.0 kHz in the non-roved simulation at 30 dB re: threshold for each model (left) and ratio of behavioral estimates of FDLs at 8.5 kHz and 2.0 kHz from various studies (right). Simulated FDLs were interpolated using LOESS while behavioral FDLs were linearly interpolated on log-log coordinates.

Ideal-observer predictions for pure-tone frequency discrimination are shown in Fig 7. The predicted FDLs generally align well with those from previous work using similar methods [27,28,56]. All-information FDLs are best near 2 kHz and then steadily degrade as the tone frequency increases (Fig 7A) due to degraded coding of TFS. However, the rate at which performance degraded with increasing frequency varied between the underlying nerve models (Fig 7C). For example, between 2 and 8.5 kHz, the Verhulst et al. [55] model predicted a factor of 5 decrease in performance, whereas the Zilany et al. [52] model predicted a factor of 25 decrease in performance, and the Heinz et al. [27] model predicted a factor of 17 decrease in performance. The onset and slope of the degradation in performance with increasing frequency in each model was directly related to the cutoff frequency and slope of phase-locking roll-off in each model’s nerve fibers (Fig 7B and S1 Text). Higher levels generally resulted in lower (better) thresholds for the all-information model. As demonstrated quantitatively over a wider range of levels in prior work [27], this trend is consistent with trends in behavioral data for frequency discrimination in quiet [57].

In contrast to the all-information observer, the rate-place observer predicted thresholds that were poorer overall and were relatively flat, or improved slightly with frequency, as expected based on sharper tuning at high frequencies (Fig 7A and 7B and S1 Text). Changes in level over the tested range appeared to have minimal impact on the rate-place observer (except at the highest frequencies in the Zilany et al. [52] model, where increased levels resulted in somewhat poorer performance). At much higher levels, rate-place thresholds degraded significantly (S3 Text). The poorer performance of the rate-place observer at high levels is not consistent with behavioral data for pure-tone frequency discrimination in quiet, which shows improved performance at high levels [57]. Consistent with expectations, performance of the rate-place observer was correlated with the sharpness of tuning in the underlying auditory-nerve model, with sharper tuning resulting in better FDLs (Fig 7B). The Zilany et al. [52] and Verhulst et al. [55] models had slightly better rate-place thresholds than the Heinz et al. [27] model, consistent with these models having sharper peripheral tuning, particularly at high frequencies.

Consistent with behavioral data [59] and the simulations of Heinz et al. [27], level roving (i.e., randomizing the level from stimulus to stimulus) had little impact on frequency discrimination for either the all-information or rate-place observers (S2 Text). In contrast, phase randomization (i.e., randomizing the starting phase from stimulus to stimulus) appeared to impair the all-information observer by a small but consistent amount (approximately a factor of two) at least up to approximately 10 kHz (S2 Text). This increase in thresholds by a factor of two closely matches the theoretical predictions made by Siebert [28] but is not observed in humans, who are insensitive to the starting phase of pure tones or the phase relationships between spectrally resolved components [60].

We compared the model results to existing behavioral data, using a function derived from multiple studies of pure-tone frequency discrimination [58]; this function’s FDLs were always larger (poorer) than the predictions of both the rate-place and all-information observers, suggesting that both models contained sufficient information to explain human behavior. However, the dependence of FDLs on frequency was better captured by the all-information model, as shown by the black curves in Fig 7A, which were scaled down by nearly three orders of magnitude in order to more closely match the absolute values of the all-information observer. In addition, we extracted FDLs from four individual behavioral studies that tested a sufficiently wide range of frequencies [21,22,36,38] and then estimated the FDL ratio between 8.5 kHz and 2 kHz for each study (Fig 7C; see Materials and Methods for details). Using the ratio of two FDLs emphasizes the effect of frequency in each dataset rather than absolute differences in FDLs between datasets or between behavioral data and the ideal-observer thresholds.

Both the behavioral data and the computational results showed a wide range in ratios of performance between 8.5 kHz and 2 kHz. Some insights can nevertheless be gained. Notably, with the exception of the Moore and Ernst [21] results, the ratios from the behavioral studies are essentially bound below by the all-information observer predictions from the Verhulst et al. [55] model (which has the smallest ratio between 8 kHz and 2.5 kHz and the slowest rolloff of phase locking) and above by the Zilany et al. [52] model (which has the largest ratio between 8 kHz and 2.5 kHz and the fastest rolloff of phase locking). In other words, despite variations in both the behavioral results and model predictions, the degradation in behavioral performance with increasing frequency is in relatively good quantitative agreement with trends observed in optimal frequency discrimination.

### F0 discrimination

**Fig 8:**
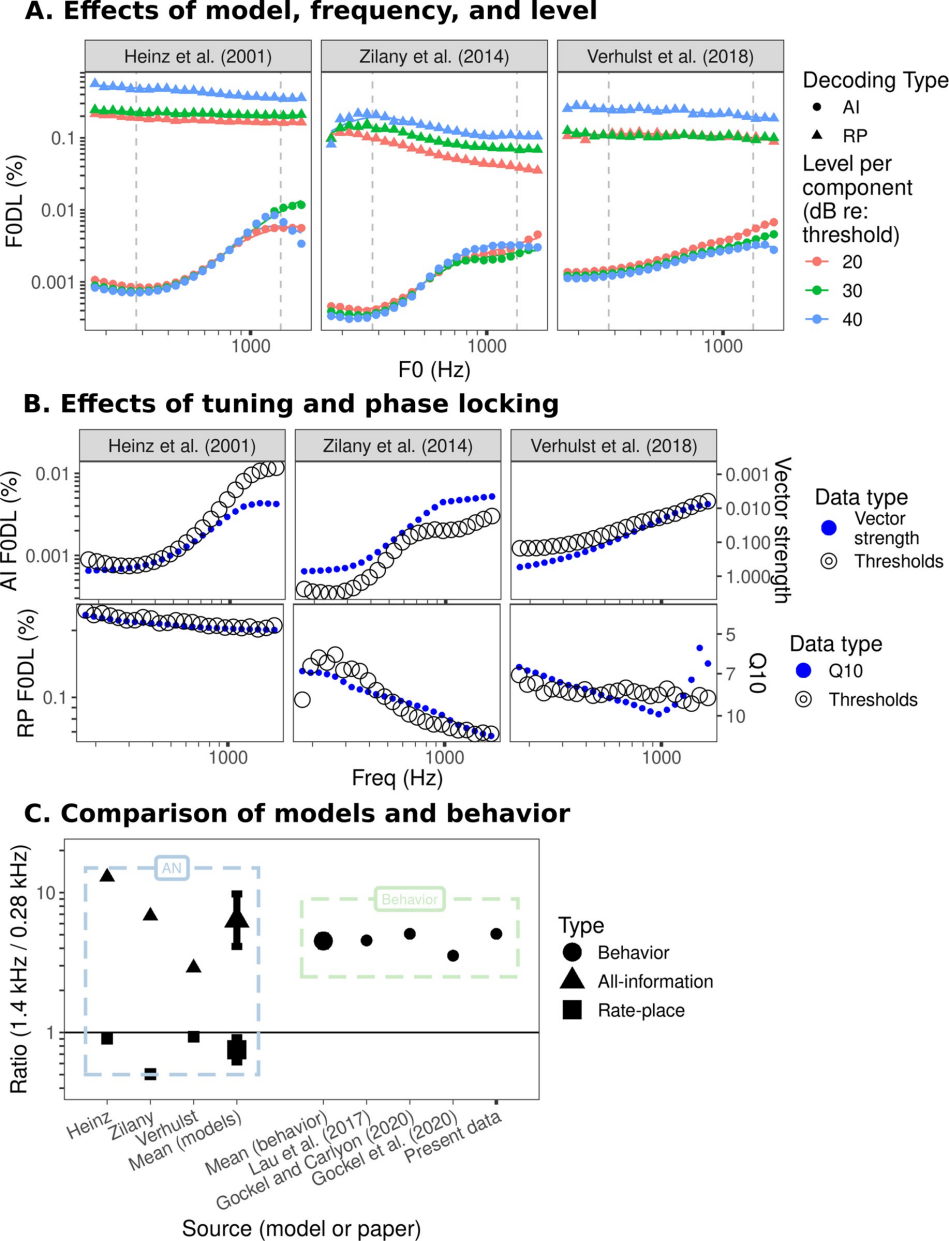
Ideal-observer predictions for F0 discrimination. Results of the F0 discrimination simulations. (A) Simulated F0DLs versus F0 of the ISO HCT stimulus in each auditory-nerve model. Simulations in this panel include no parameter roving. Points indicate the simulated F0DLs at a particular F0 while lines indicate a locally estimated scatterplot smoothing (LOESS) fit to the simulated F0DLs. (B) Simulated all-information F0DLs and vector strength (top row) and simulated rate-place F0DLs and Q10 (bottom row) versus frequency with a double y-axis. To choose the warping on the y-axis for vector strength and Q10, linear models were fit to predict log-transformed F0DLs as a function of log-transformed reciprocals of vector strength or Q10 for the all-information F0DLs or rate-place F0DLs, respectively. The fitted regression equations were then used to warp the y-axes. In other words, we warped the y-axes for vector strength and Q10 to maximize overlap with the model predictions (across all three models) in order to visually demonstrate the relationship between vector strength and Q10 and the simulated F0DLs (C) Ratio of simulated F0DLs at 1.4 kHz and 0.28 kHz in the non-roved simulation at 30 dB re: threshold for each model (left) and ratio of behavioral estimates of F0DLs at 1.4 kHz and 0.28 kHz from various studies (right). Simulated F0DLs were interpolated using LOESS while behavioral F0DLs were linearly interpolated on log-log coordinates.

Ideal-observer predictions for F0DLs with harmonics 6–10 are shown in Fig 8. The all-information observer predicted that F0 discrimination should be best for F0s in the range of 200–500 Hz (with the precise range depending on the underlying auditory-nerve model) and then steadily worsen with increasing F0 before plateauing around 600–1000 Hz (Fig 8A), at least in the Heinz et al. [27] and Zilany et al. [52] models. For the present stimuli, the 6th harmonic was the lowest present, so that an F0 of 600 Hz corresponded to a lowest harmonic of 3600 Hz, whereas an F0 of 1000 Hz corresponded to a lowest harmonic of 6000 Hz. The model fibers do not phase lock strongly to frequency components above about 4 kHz (S1 Text); thus, this pattern of results is suggestive of a transition from more accurate TFS-based coding at lower F0s to less accurate envelope-based coding at higher F0s in the all-information observer (see no-masker simulations in Fig 5C).

As expected, and consistent with the results for frequency discrimination, the rate-place observer thresholds were higher than the all-information observer thresholds. Nevertheless, as with the pure-tone FDL predictions, the rate-place thresholds were still better than the average behavioral thresholds, suggesting that, in principle, sufficient rate-place information is available at the level of the auditory-nerve to account for the *accuracy* of behavioral F0 discrimination at both low and high frequencies. Performance for the rate-place observer was generally flat with frequency or slightly improved with higher frequencies, depending on the exact model in question. These inter-model differences are a direct result of differences in peripheral tuning between the models (Fig 8B and S1 Text). The rate-place observer tended to be more sensitive to changes in level than the all-information observer (Fig 8A). Generally, rate-place thresholds were poorer at higher levels, consistent with the fact that higher levels degrade representations of stimulus harmonics in the average rate responses of auditory-nerve fibers [61]. At much higher levels, rate-place thresholds worsened significantly, consistent with saturation of the simulated high-spontaneous-rate fibers at high levels (S3 Text). There is some evidence that F0 discrimination (in noise) worsens with increasing level [62]; however, this effect was observed at higher levels behaviorally than those tested in the present model simulations.

Both the all-information observer and the rate-place observer models were affected by parameter roving. Level roving typically had a negative impact on the rate-place observer at higher frequencies but little impact on the all-information observer (S2 Text). In comparison, phase randomization (i.e., randomizing the relative phases of the components from stimulus to stimulus instead of always synthesizing components in sine phase) had no impact on the rate-place observer but had a negative impact on the all-information observer (S2 Text). Generally, across a fairly wide range of levels and frequencies, phase randomization elevated all-information thresholds by a factor of two, consistent with the predictions of Siebert [28] and the frequency discrimination results. In behavioral data, phase randomization only affects thresholds when all the stimulus components present are unresolved, as expected [31,32].

To compare the model results to behavioral data, we extracted discrimination thresholds from three behavioral studies that tested the same F0s as in our study [36–38] and then calculated the ratio between F0 discrimination thresholds for F0s of 1.4 kHz and 0.28 kHz for each study as well as the present study (Fig 8C; for the present study, data was pooled across Experiment 1a and Experiment 1b, see Materials and Methods for details). In contrast to the varying stimuli and methods of the selected pure-tone frequency discrimination studies, the selected F0 discrimination studies all used essentially the same stimuli and methods. The ratios between F0 discrimination thresholds at 1.4 kHz and 0.28 kHz for each study ranged from approximately 3 [36] to approximately 5 [37]. These values are within a range that can be plausibly explained by the degradation of the all-information observer with increasing frequency, with the same ratio for the all-information observer ranging from approximately 3 for the Verhulst et al. [55] model to over 10 for the Heinz et al. [27] model. In contrast, the rate-place observer model again predicted threshold ratios of around 1, which is outside the range observed in behavioral data.

As was the case for frequency discrimination, the all-information observer provided the best overall match to human data in terms of *relative* performance at low and high frequencies. The plateau in the predicted F0 discrimination thresholds of the all-information observer above about 600 Hz in the Heinz et al. [27] and Zilany et al. [52] models (in contrast to the progressive degradation of predicted pure-tone frequency discrimination thresholds at high frequencies; Figs 7 and 8) suggests that, at higher F0s once phase locking to TFS becomes unavailable, the all-information observer relies on temporal cues produced by peripheral interaction between adjacent harmonics rather than phase locking to TFS at the component frequencies (Fig 1B).

The correspondence between all-information trends and human behavior could be interpreted as support for the use of temporal-envelope cues at high F0s by humans. However, as discussed earlier, several lines of evidence from pitch psychophysics pose challenges to this interpretation. Specifically in the case of the high-F0 HCTs with harmonics in the range of the 6^th^ to the 10^th^, pitch perception is robust to manipulations in which the even and odd harmonics are presented to different ears (which would decrease modulation depth and double the modulation rate in each ear [38]) but is impaired by harmonicity manipulations in which the frequencies all components are shifted up by a constant amount in Hz (which preserves modulation rates while rendering the tone inharmonic [38,39]). Although beyond the scope of this paper, auditory-nerve simulations and ideal-observer analysis could help further clarify our understanding of these conditions. More generally, for HCTs composed of unresolved harmonics and amplitude-modulated noises (for which pitch is thought to be exclusively conveyed by temporal-envelope cues), humans are not able to achieve accurate pitch discrimination for F0s (or modulation rates) beyond about 600–700 Hz [40,42]. These lines of evidence seem to rule out a class of simple models wherein listeners compare temporal-envelope rates to discriminate F0 in the present task. However, there are several notable differences between the present HCT stimuli and the stimuli used to probe temporal-envelope pitch (unresolved HCTs and modulated noises) that are worth considering further. First, whereas for unresolved HCTs and modulated noises the only reliable pitch cue is the temporal-envelope rate, the present HCT stimuli consist of partially resolved harmonics, which provide rate-place cues in addition to any temporal cues elicited by peripheral interaction between stimulus components. Second, whereas for unresolved HCTs and modulated noises, all auditory-nerve fibers tuned to the stimulus should have strongly modulated responses, in the present HCT stimuli, modulation power varies substantially over the range of CFs, with deeper modulations in channels tuned between stimulus components (Figs 1 and 5). As a result, for unresolved HCTs or modulated noises, the auditory system is relegated to comparing temporal-envelope modulation rates to perform discrimination. In contrast, for the present HCT stimuli, the auditory system could derive F0 estimates by combining information provided by average ANF rates (the excitation pattern) with information provided by the distribution of envelope modulation power across CFs (the so-called “fluctuation profile”; [63]). Recent modeling studies suggest that such a strategy, based on decoding of simulated fluctuation profiles at the level of the midbrain, can in principle account for behavioral F0 discrimination in stimuli similar to the present low-frequency HCT stimuli [64] as well as performance in other psychoacoustical tasks [65]. At the same time, these prior modeling studies have focused on midbrain neurons tuned to modulations on the order of around 100 Hz, while stimulus-envelope modulations in the present high-frequency HCT stimuli are at much higher rates (> 1 kHz) where sensitivity to amplitude modulations is thought to be limited [66–68]. Further investigation will be needed to determine whether the fluctuation-profile approach can be successfully extended to our high-frequency stimuli and reconcile the trends in the ideal observer with what is known from pitch psychophysics.

### Effect of maskers

In theory, the ideal-observer model could be applied to the Experiment 1 GEOM stimulus and the Experiment 2 DBL stimulus to investigate how HCT maskers affect F0 discrimination. However, this was not done for the present analyses for reasons discussed in Supporting Information (S4 Text). The primary issue was that preliminary attempts at modeling these conditions usually resulted in the ideal observer performing *better* in the presence than in the absence of a masker (Fig A in S4 Text). Although initially surprising, further consideration of the nature of the ideal observer makes it clear why this could happen. As shown previously [27], the lower bound on the precision of estimating a stimulus parameter in our model, as specified by the Cramér-Rao lower bound, is equivalent to the performance achieved by a maximum likelihood estimator (at least for the one-parameter case). The maximum likelihood estimator examines each spike in the auditory-nerve response and determines whether it is more probable that the spike came from a baseline interval (*f*) or an incremented interval (i.e., *f + Δf*) and then combines those probabilities across all observed spikes. Intuitively, this means that the observer has perfect templates of what the baseline and incremented responses should look like and compares the response against those templates according to an optimal rule. In the case of stimuli containing maskers, this means that the ideal observer can exploit *any* feature of the responses in decoding. As observed previously for noise maskers [69], interactions between targets and maskers that would never be usable by human observers (because they are completely unpredictable from trial to trial) can be exploited by the ideal observer, leading to unrealistic thresholds. To resolve these limitations of the present ideal-observer model, future work could explore the application of quasi-ideal observers [69,70], deep neural network models [71], or other modeling frameworks to our stimuli to better understand how F0 cues may be more realistically decoded in HCT-mixture stimuli.

## Discussion

Our results provide novel insights into F0 coding at high frequencies and in the presence of competing harmonic sounds. Experiment 1 replicates prior findings to confirm that listeners can accurately discriminate changes in the F0 of HCTs composed of spectrally resolved components even when those HCTs are composed of frequencies entirely beyond 8 kHz [36–38], and extends them to demonstrate that listeners can maintain accurate discrimination even in the context of a single spectrally overlapping concurrent HCT masker. However, consistent with prior results, performance in all conditions of Experiment 1 was worse at high frequencies than at low frequencies. Experiment 2 revealed that this high-frequency deficit in performance was compounded by the addition of two spectrally overlapping concurrent HCT maskers. In this experiment, listeners were often unable to perform F0 discrimination at high frequencies in the presence of the two maskers, even at large TMRs, whereas at low frequencies many listeners were able to do so at TMRs only slightly above 0 dB. Qualitative visualizations of simulated auditory-nerve responses to our stimuli raised the possibility that resolved target harmonics were available at high frequencies in masked conditions in Experiment 1 but not in Experiment 2, potentially explaining differences in the results of these two experiments. However, these visualizations also highlighted the complexities inherent in interpreting behavioral responses to HCT-mixture stimuli. Thus, our behavioral results collectively provided limited support for the notion that accurate high-frequency F0 discrimination is based on a rate-place code.

We next implemented a neural ideal-observer model. This model highlighted a key difference between optimal frequency discrimination and F0 discrimination as a function of frequency; whereas optimal frequency discrimination based on temporal information should continue to degrade with increasing frequency until it eventually matches performance based on average rate cues alone (Fig 7A), the presence of envelope fluctuations in CFs tuned between stimulus harmonics (or to unresolved stimulus harmonics) results in optimal F0 discrimination that plateaus above a certain frequency even as phase locking continues to degrade (Fig 8A). If all the information available in responses is used optimally (all-information ideal observer), predicted thresholds show a dependency on frequency and F0 that is similar to that found in humans, but predicted thresholds are several orders of magnitude better than human thresholds. If instead only time-averaged information is considered (rate-place ideal observer), overall threshold predictions are more comparable in magnitude to human thresholds but do not show the same dependencies on frequency or F0. In other words, for the all-information thresholds to explain human data, later processing stages must introduce coding inefficiencies or noise that are large and uniform across the tonotopic range. In contrast, for the rate-place thresholds to explain human data, later processing stages must introduce relatively little noise, and that noise must be greater at high frequencies or F0s.

In summary, our results suggest that human complex pitch perception at low and high frequencies is not principally limited by the information available in the auditory nerve. Further research integrating psychophysical and physiological data via computational modeling will be needed to understand how limitations originating in non-peripheral loci contribute to complex pitch perception in humans. A wide range of modeling frameworks, including ideal-observer analysis [27,28], template-based models [64,65], and neural-network models [71], may all contribute to this endeavor in meaningful ways. For example, whereas neural-network models excel at shedding light on the links between statistical patterns in natural stimuli and complex pitch perception, ideal-observer models can help understand tasks (such as the present high-F0 pitch discrimination) that employ stimuli with low prevalence among natural sounds (i.e., stimuli that are sparsely represented in the naturalistic training data needed for deep neural networks). An improved understanding of pitch coding resulting from these investigations may aide in the quest to restore normal pitch perception for listeners with hearing loss and cochlear implants [6,72,73].

## Materials and methods

### Ethics statement

The behavioral data collected for this study was collected according to protocols approved by the University of Minnesota Institutional Review Board. Informed consent was obtained from participants in written form prior to initiation of the experimental protocol.

### Behavioral data collection

#### Equipment

All stimuli were presented to listeners diotically over HD650 headphones (Sennheiser, Old Lyme, CT) in sound-attenuating booths via a Lynx E22 sound card (Lynx Studio Technologies, Costa Mesa, CA) at a sampling rate of 48 kHz. Listeners completed the experiment via a graphical user interface implemented in MATLAB (The Mathworks, Natick, MA) via the AFC framework [74].

### Participants

Participants in the present experiments were all students at the University of Minnesota with audiologically normal hearing. Of the 36 participants that passed the screening procedures described below (29 female, 7 male), the mean age was 22.8 years (SD = 1.9, minimum = 20, maximum = 28). Participants were recruited either through a Department of Psychology research participant pool or through an in-house participant database. Participants were paid for their participation.

### Procedures

All experiments began with a three-stage screening process. The first stage consisted of a standard audiogram to ensure normal-hearing status (i.e., audiometric thresholds ≤ 20 dB hearing level [HL] at octave frequencies from 0.25 to 8 kHz). The second stage consisted of measuring detection thresholds for pure tones embedded in the same broadband TEN used in the main experiments. The purpose of this stage was to ensure that participants would be able to hear all the target stimuli in the presence of the masking noise during the experiments. Detection thresholds were measured using a two-interval two-alternative forced choice (2I2AFC) method combined with a 3-down 1-up staircase procedure [75]. Both intervals contained the TEN and one interval, chosen at random on each trial, contained the target tone. The participants’ task was to select the interval containing the tone. The tones were 350 ms in duration, including 20-ms raised-cosine onset and offset ramps. The noise was 500 ms in duration, including with 20-ms raised-cosine onset and offset ramps, and the tones were temporally centered in the noise. The noise had a level of 40 dB SPL in the estimated ERB of the human auditory filter centered on 1 kHz. The adapted variable was the rms level of the target in dB SPL. The starting value of target level was 55 dB SPL. The initial step size of the adaptive procedure was 3 dB. After two reversals in the direction of the adaptive procedure, the step size was reduced to 2 dB. After another two reversals this was reduced to 1 dB (the smallest step size used). The procedure was terminated after 6 reversals at the smallest step size and the threshold was defined as the mean of the target levels at the last six reversals. Tone frequencies of both 14 kHz and 16 kHz were tested. In order to pass this stage, participants needed thresholds better than 45 dB SPL at both frequencies, averaged across three runs per frequency. This value was 2 dB lower than the lowest possible presentation level of individual harmonics in the experimental stimuli, and so it was thought that participants who could pass this criterion should be able to detect the stimuli during the experiment. Participants who failed this stage did not complete any other part of the experiment. Of the 79 participants who attempted the audibility screening, 44 passed (pass rate = 56%) and were invited to participate in following stages of screening.

The third stage consisted of measuring F0DLs for the two nominal F0s tested in the experiments described below (280 Hz and 1400 Hz). The purpose of this stage was to ensure that participants had the ability to accurately label the direction of a change in F0. The F0DLs were measured using a 1I2AFC task and a 3-down 1-up adaptive staircase procedure. In each trial, three isolated HCTs with the same F0 (the reference F0) were presented in sequence as a precursor, followed by a target HCT with a different F0 (the test F0) with a higher or lower F0 than the precursor tones (selected at random on each trial with equal probability). All the precursor and target tones were separated from each other by 50-ms silent intervals. The listener’s task was to judge whether the target F0 was higher or lower than the reference F0. Visual feedback was provided immediately after each trial. The starting value of DF0 (the difference between the reference and target F0s) was 10% of the lower F0. The initial step size was a change in DF0 by factor of 2. After two reversals this was adjusted to a factor of 1.41. After another two reversals this was adjusted to a factor of 1.19 (the smallest step size used). The procedure was terminated after 6 reversals at the smallest step size and the threshold was defined as the geometric mean of the DF0 at the last six reversals. The maximum permitted DFO was approximately 30%; if a larger DF0 was called for by the adaptive procedure on more than 6 consecutive trials then the run was terminated early and no threshold was recorded. The reference and target F0s were geometrically centered around an F0 selected randomly on each trial from a uniform distribution of either 280 ±10% Hz (low-frequency condition) or 1400 ±10% Hz (high-frequency condition). Each tone was 350 ms in duration, including 20-ms raised-cosine onset and offset ramps. The tones were composed of harmonics 6–10, presented in random phase (Experiment 1a and Experiment 2) or presented with all harmonics up to the Nyquist frequency in sine phase and then zero-phase bandpass filtered with a 12^th^-order Butterworth filter with cutoffs at 5.5 to 10.5 times the nominal F0 (Experiment 1b). The level of each harmonic was 50 dB SPL. In order to pass this stage, participants were required to obtain thresholds better than 6% and 12% for the low- and high-F0 conditions, respectively, averaged across three runs per nominal F0. Participants who failed this stage were given two additional chances to pass the screening under an identical procedure. Participants who passed on any attempt moved on to complete the experiment while participants who failed all attempts did not complete any other part of the experiment. Of the 44 participants who attempted the pitch screening, 36 passed (pass rate = 82%) and went on to complete one or more experiments.

#### Experiment 1

Experiment 1 measured F0DLs using the same procedure as described above for the F0DL screening. The reference and target F0s were geometrically centered around an F0 selected randomly on each trial from a uniform distribution of either 280 ±10% Hz (low-frequency condition) or 1400 ±10% Hz (high-frequency condition). In Experiment 1a, the tones were composed of harmonics 6–10 of their F0 synthesized in random phase. In Experiment 1b, the tones were synthesized with all harmonics of their F0 up to the Nyquist frequency in random phase and then zero-phase bandpass filtered with a 12^th^-order Butterworth filter with cutoffs at 5.5 and 10.5 times the nominal F0 (either 280 or 1400 Hz). The level of each harmonic (before filtering) was roved independently by ±3 dB (uniform distribution) around the nominal level of 50 dB SPL. These stimulus parameters were selected to satisfy two key criteria. First, by using the same low harmonic numbers for both nominal F0s, harmonic resolvability was comparable across the nominal F0s. Second, by ensuring that the lowest frequency component of the target was almost always above 8000 Hz when the nominal F0 was 1400 Hz, phase locking cues were likely limited for the higher nominal F0 while remaining intact for the lower nominal F0 [23,29]. The tones on each trial were embedded in TEN, which was newly generated in each trial, began 75 ms before and ended 75 ms after the tones, and had 20-ms raised cosine-ramps. The TEN noise had a level of 40 dB in the ERB of the auditory filter centered on 1 kHz. This noise was added to ensure that any distortion products generated by the tones were not audible to listeners [39].

Two masker types were tested in Experiment 1. The first, ISO, consisted of only the precursor and target presented in isolation. The second, GEOM, consisted of the precursor presented in isolation and the target presented simultaneously with a single concurrent and spectrally overlapping complex tone masker. The masker had an F0 which was the geometric mean of the reference and target F0s. In Experiment 1a, the masker was generated in a fashion identical to that of the target except that it included two additional harmonics (5 and 11). In Experiment 1b, the masker was generated in a fashion identical to that of the target except that the bandpass filter was slightly broader, extending from 4 to 12 nominal F0 rather than only 5.5 to 10.5 times the nominal F0. Thus, in both cases, the masker bandwidth always exceeded the target bandwidth. For both Experiments 1a and 1b, seven runs of each condition (ISO Low, ISO High, GEOM Low, and GEOM High) were measured for each listener in random order.

Experiment 1 took approximately 2–3 hours to complete, and most listeners completed it in one or two sessions lasting no more than two hours each. In total, 36 listeners took part in some portion of Experiment 1. Specifically, 12 listeners took part in only Experiment 1a, 16 listeners took part in only Experiment 1b, and 8 listeners took part in both Experiments 1a and 1b.

#### Experiment 2

Experiment 2 measured TMRs for a fixed interval size at the 79.4% correct point via a 2I2AFC task with a 3-down 1-up adaptive staircase procedure. The only masker type tested was the DBL masker, in which two concurrent and spectrally overlapping HCT maskers were presented simultaneously with the target tone, following the precursor with the three reference tones. One masker had an F0 5.25 to 7.25 semitones (uniform distribution) higher than the nominal F0 while the other masker had an F0 –5.25 to –7.25 semitones (uniform distribution) lower than the nominal F0. The targets and maskers were synthesized as in Experiment 1b. The interval sizes used in Experiment 2 were determined individually for each listener based on their performance in Experiment 1b. For each nominal F0, both 1.5 and 2.5 times the listener’s F0DL were tested. Only listeners for whom 2.5 F0DL at both nominal F0s was less than 2.6 semitones participated in Experiment 2 (to ensure that the reference F0 would always be further from the masker F0s than from the target F0 for all listeners and interval sizes). The starting value of TMR was 0 dB. The initial step size was 3 dB. After two reversals this was adjusted to a step size of 2 dB. After another two reversals this was adjusted to a step size of 1 dB (the smallest step size used). This procedure was terminated after six reversals at the smallest step size and the threshold was defined as the mean of the TMRs at the last six reversal points. As in Experiment 1, listeners completed seven adaptive runs of each condition. Experiment 2 took approximately 2–3 hours to complete, and most listeners completed it in one to two sessions. A total of 11 listeners completed Experiment 2.

### Behavioral data analysis

#### Experiment 1a

A total of 20 participants participated in Experiment 1a, but only those who maintained average performance levels above the screening criteria in the testing phase were included in the present data analysis (*n* = 13). Out of a total of 348 measured adaptive runs, five adaptive runs were terminated early according to the rules described in the methods above. Thresholds were not recorded for these runs. Data from these terminated runs were not used in the present data analysis. A generalized linear mixed-effects model (GLMM) was used to analyze the results from Experiment 1a with the thresholds expressed in units of 10log_10_(%). The fixed effects included the masker (ISO or GEOM) and the nominal F0 (280 or 1400 Hz) as well as their interaction, while the random effects included random intercepts and slopes for listener (i.e., a maximal random effects structure) [76]. The model was implemented using the R programming language [77] and the lme4 package [78] via penalized maximum likelihood estimation. Before proceeding with analysis and interpretation of the model, diagnostic checks by visual inspection of QQ plots, scale-location plots, a plot of standardized residuals versus fitted values, and of standardized residuals versus experimental conditions were made. Although the distribution of individual means appeared to be non-normal in some cases (Figs 2 and 3), the diagnostic plots suggested that nevertheless residual variance in the model was approximately normal and homoscedastic.

The model was analyzed in two ways. First, the significance of the fixed effects was examined by likelihood ratio *F*-tests in a Type III analysis of variance (ANOVA). The *F*-tests was calculated using the Kenward-Rogers approximation for the denominator degrees of freedom via the car [79] and pbkrtest packages [80]. Second, the significance of linear contrasts of model coefficients was examined by *F*-tests via the phia package [81]. All *p*-values in Experiment 1a were jointly corrected using the Holm-Bonferroni method. Corrected *p*-values were reported and were compared against a criterion of *θ* = 0.05 to assess statistical significance.

#### Experiment 1b

The same general analysis framework used in Experiment 1a was repeated to analyze the data from Experiment 1b. A total of 24 participants participated in Experiment 1b, but only those who maintained average performance levels above the screening criteria in the testing phase were included in the present data analysis (*n* = 17). Out of a total of 475 measured adaptive runs, 10 adaptive runs were terminated early according to the rules described in the methods above without recording a threshold. Data from these terminated runs were not used in the present data analysis. The fixed and random effects structures from used in the model for Experiment 1a were repeated here, and the same model fitting and analysis approach was also repeated here.

#### Experiment 2

The same general analysis framework used in Experiment 1 was repeated to analyze the data from Experiment 2. A total of 12 participants completed Experiment 2 and were included in the present data analysis. The fixed effect included the interval size (1.5 or 2.5 F0DL) and the nominal F0 (280 or 1400 Hz) as well as their interactions, while the random effects included random intercepts and slopes for listener (a maximal random effect structure). The same model fitting and analysis approach used for Experiment 1 was repeated here to analyze the present model.

### Computational model

We used three previously published auditory-nerve models to conduct our simulations of auditory-nerve firing rates. The use of multiple nerve models affirmed that the key trends were not artifacts of assumptions or features that were specific to a particular model. First, we used the auditory-nerve model described in Heinz et al. [27]. Although simpler and less accurate than the other nerve models we tested, this model had the advantage of being much faster than the other models and it allowed for a more direct comparison between the present results and previous auditory-nerve ideal-observer results based on this model [27,56]. This model was implemented in Python and run at a sampling rate of 1250 kHz (higher than the other models to reduce distortion at high stimulus frequencies and levels). Second, we used the auditory-nerve model of Zilany et al. [52]. The model was run in an adapted version of the Python interface to the original C implementation of the model provided by the cochlea package [82]. The model was run with parameters designed to replicate the human peripheral tuning estimates of Shera et al. [51] at a sampling rate of 200 kHz (for the basilar-membrane and inner-hair-cell stages) and 50 kHz (for the synapse stage). Finally, we used the auditory-nerve model of Verhulst et al. [55]. The model was run via the authors’ published Python code at a sampling rate of 300 kHz.

#### Excitation pattern and autocorrelogram visualizations

Fig 4 shows excitation patterns at low frequencies and at high frequencies for both the ISO and GEOM stimuli. Each excitation pattern was constructed by simulating instantaneous-rate responses at 200 CFs ranging from 4F0 to 12F0 for high-spontaneous-rate and low-spontaneous-rate fibers from the Zilany et al. [52] model. Tones were set to 50 dB SPL per component and TEN was included at a level of 40 dB SPL in the ERB centered on 1 kHz. Each excitation pattern reflects the average of 10 simulations over fresh samples of level roving (± 3 dB per component, independent) and TEN. The simulations were conducted at a sampling rate of 100 kHz (for the basilar-membrane and inner-hair-cell stages) and 20 kHz (for the synapse stage).

Fig 5 shows autocorrelograms for the DBL stimulus and Fig 6 shows excitation patterns for the DBL stimulus. Autocorrelograms for Fig 5 were calculated based on instantaneous-rate responses from a population of high-spontaneous-rate auditory-nerve fibers using the Zilany et al. [52] nerve model. The stimulus was the DBL stimulus with target levels set to 50 dB SPL per component, masker levels set to 0, 5, or 10 dB below the target levels (or no masker), and TEN at a level of 40 dB SPL in the ERB centered on 1 kHz. One masker F0 was set to 5.5 ST below the target F0 and the other masker F0 was set to 6 ST above the target F0. Simulations were conducted for CFs ranging from 4F0 to 12F0 with F0 set to 280 Hz or 1400 Hz and then the autocorrelation was calculated at each CF. The simulations were conducted at a sampling rate of 200 kHz. Excitation patterns for Fig 6B were generated as in Fig 4, except the stimulus used was the DBL stimulus used for the autocorrelograms in Fig 5. Both low-spontaneous-rate fibers and high-spontaneous-rate fibers were simulated. The target F0 was set to 280 Hz or 1400 Hz. The simulations were conducted at a sampling rate of 100 kHz.

#### Threshold estimation

The effective level of stimulation in the present auditory-nerve models significantly altered predicted thresholds. This effect complicated interpretation of the relationship between estimated thresholds and stimulus frequency and comparisons between the models, as the effective presentation level varied with frequency due to differences in simulated middle ear transfer functions and other factors in each model. To ameliorate these issues, we estimated the rate-level threshold as a function of CF for each auditory-nerve model we tested and used this information to present stimuli at a constant level relative to these thresholds across stimulus frequency. For each auditory-nerve model, we simulated the average firing rate in response to 100 ms pure tones presented at each of 25 frequencies spaced logarithmically from 0.2 to 20 kHz for a high-spontaneous-rate auditory-nerve fiber tuned to the same frequency. This process was repeated at 25 levels spaced linearly (in dB) from -10 to 40 dB SPL. Next, the rate-level function at each frequency was linearly interpolated and the minimum level required to evoke a firing rate 5% above the spontaneous firing rate was recorded as the rate-level threshold for that CF. Finally, the rate-level thresholds at were linearly interpolated as a function of CF and saved to disk for later use in adjusting presentation levels in the simulations.

#### Frequency discrimination simulations

Ideal-observer predictions were generated for discriminating the frequency of pure tones, as in Heinz et al. [27]. Although our study did not measure FDLs, the simulated FDLs provide a way of validating our ideal-observer analysis framework against prior results and provide important context to the more complex F0 discrimination results. For each tested frequency, firing rates were simulated for 40 CFs distributed logarithmically from 0.5 to 1.5 times the frequency. All the simulated fibers were high-spontaneous-rate fibers. It was assumed that 72 fibers with nearby CFs shared the firing rate pattern at each simulated CF. Thus, the 40 CFs represented an approximation to an underlying population of approximately 3000 high-spontaneous-rate fibers in the auditory nerve tuned near the stimulus frequency. These values were selected under the assumption that, from a total population of 30,000 auditory-nerve fibers, approximately 60% would be high-spontaneous-rate fibers, 20% would be medium-spontaneous-rate fibers, and 20% would be low-spontaneous-rate fibers. Increasing the number of CFs and decreasing the number of fibers per CF would result in a more accurate sampling of the response over the range of CFs and therefore a more accurate estimate of the optimal threshold; however, it would also result in a corresponding increase in the computational load of the simulations. The choice of 40 CFs was thus a compromise between computational resources and accuracy (although increases in the number of simulated CFs in prior testing did not appear to significantly change estimated thresholds beyond a certain point).

The pure tone stimuli used in the simulations were 100 ms in duration, including had 10 ms-raised-cosine ramps. Twenty-four frequencies spaced logarithmically from approximately 1.4 to 14 kHz (corresponding to the frequencies of the 8th harmonic in the F0s tested below) were tested. Stimuli were presented to the models at nominal levels of 20, 30, and 40 dB re: threshold. In the simplest simulations, the level and starting phase of the pure-tone stimulus was fixed (i.e., Eq 2 was used, see below). A starting phase of 0 degrees was used in this case. Intuitively, this simulation can be conceptualized as one in which the ideal observer “knows” the starting phase of the stimulus and that starting phase is always 0 degrees. In some simulations, however, either the level or the starting phase of the pure-tone stimulus was treated as a random variable (i.e., Eq 6 was used, see below). These types of simulations are referred to using the terms “level roving” and “phase randomization”, respectively. In the simulations, level was distributed uniformly in a ±3 dB range around the nominal level and phase was distributed uniformly over a 360-degree range (matching the roving/randomization applied to individual components in the behavioral experiments). However, when evaluating the model equations, level was treated as if it were normally distributed with a standard deviation of 6 dB, while starting phase was treated as if it were normally distributed with a standard deviation of 360 degrees. Normal distributions were used to approximate the uniform distributions used in the behavioral experiments to ensure the Fisher information would be well defined. The standard deviations were selected to match the approximation used in prior work [56]. Intuitively, these simulations can be conceptualized as ones in which the ideal observer does not know the value of the randomized parameter (level or phase) beforehand but instead infers it in the same way that it infers the frequency of the stimulus (i.e., optimally at the limit defined by the Cramér-Rao lower bound; see below).

In Fig 7, data from prior behavioral studies were included for comparison to the simulation results [21,22,36,37]. We extracted these data visually from the corresponding figures and then linearly interpolated each dataset on a log-log axis in order to estimate the ratio between FDLs at 8 kHz and 2.5 kHz in each study. From Moore [22], data from the 200 ms condition were selected. From Moore and Ernst [21], data from the 20 dB SL condition were selected. From Gockel et al. [36], data from the short diotic (210ms) condition were selected. Additionally, we generated FDL predictions from the model of Micheyl, Xiao, and Oxenham [58]. Values of free parameters in their model were set according to their posterior mean estimates, the sensation level was set to 25 dB, and the duration was set to 200 ms.

#### F0 discrimination simulations

Ideal-observer predictions were generated for discriminating the F0 of the ISO stimulus from Experiment 1. The simulations were performed in the same way as the frequency discrimination simulations, except that firing rates were simulated for 40 characteristic frequencies (CFs) distributed logarithmically from 5 F0 to 11 F0 and that each CF was assumed to reflect the contribution from 51 nearby fibers.

The HCT stimuli used in the simulations were those from Experiment 1b, except they were only 100 ms in duration, they were presented without noise, and they were presented at a wider range of levels and F0s than in the behavioral experiment. F0s spaced logarithmically from 0.18 to 1.8 kHz were tested. Levels were set such that the nominal level per component was 20, 30, or 40 dB re: threshold at the 8^th^ harmonic. In the simplest simulations, the levels and relative phases of the stimulus components were fixed (i.e., Eq 2 was used, see below). The stimulus components were synthesized in sine phase in this case. In some simulations, however, either the levels or the relative phases of the components were treated as random variables (i.e., Eq 6 was used, see below). These types of simulations are referred to using the terms “level roving” and “phase randomization”, respectively. In the simulations, component levels were distributed uniformly in a ±3 dB range around the nominal level and relative phases were distributed uniformly over a 360-degree range (matching the roving/randomization applied to individual components in the behavioral experiments). However, when evaluating the model equations, the same approximation used for frequency discrimination above was used here.

In Fig 8, data from prior behavioral studies were included for comparison to the simulation results [36–38]. These data were extracted visually from the corresponding figures and then the ratio between F0DLs at 1.4 kHz and 0.28 kHz was calculated for each dataset. From Gockel et al. [36], we selected the thresholds for the 210-ms tones presented in diotic TEN. Because performance in the compared conditions did not differ between Experiment 1a and Experiment 1b, data from the present study used in this figure was pooled across both Experiment 1a and Experiment 1b.

#### Ideal observer

The general mathematical framework for ideal-observer analysis as applied to the auditory nerve was described first in Siebert [28], who employed the technique with a simple analytic model of the auditory nerve. Later authors applied the same approach to simulated auditory-nerve responses from more realistic computational models of the auditory nerve [27,56,70]. For convenience, the key derivation steps and insights of this analysis technique are described briefly below; additionally, a generalization of the technique for cases with an arbitrary number of stimulus parameters is provided. For a more complete treatment the reader is referred to the existing literature [27,28,56,69].

We begin by letting *r*_*i*_(*t*, *θ*) indicate the firing rate of the *i*-th auditory-nerve fiber at time *t* in response to the stimulus with parameter *θ*. We assume that ***x***_*i*_, the spike times of fiber *i* responding to our stimulus, are Poisson distributed with time-varying rate parameter *r*_*i*_. That is, ***x***_*i*_ is the stochastic spike data observed from fiber *i*, while *r*_*i*_ is the time-varying instantaneous firing rate of fiber and *i* is a deterministic function of the input stimulus (which is itself deterministic). We also assume that ***x***_*i*_ and ***x***_*j*_, for all *i* and *j* where *i* ≠ *j*, are conditionally independent given *θ*.

***X*** (the joint distribution of spike times from all fibers) contains information about *θ*, and observers of ***X*** seek to utilize that information to infer the value of *θ*. We henceforth define an ideal observer as any such observer that uses the best unbiased estimator of *θ* (in terms of having the lowest variance or, equivalently, the highest precision) to infer *θ* from ***X***. The Cramér-Rao lower bound provides an upper bound on the precision of any such estimator:

$$\frac{1}{\sigma_{\hat{\theta }}^{2}}\leq I(\theta )$$ (1)

where $\sigma_{\hat{\theta }}^{2}$ is the variance of the estimator for *θ* based on observing ***X***, and *I*(*θ*) is the Fisher information of *θ* in ***X***. As noted by Siebert [28] for our unordered spike times from a population of auditory nerve fibers ***X***:

$$\frac{1}{\sigma_{\hat{\theta }}^{2}}\leq \sum_{i}\int_{0}^{T}\frac{1}{r_{i}(t,\theta )}{\left(\frac{\partial r_{i}(t,\theta )}{\partial \theta }\right)}^{2}$$ (2)

By simulating auditory-nerve responses to the stimuli to estimate r_i_ and approximating the partial derivative with respect to θ via the finite differences method, we can arrive a computational estimate of the inequality [27,56]. This estimate can then be transformed into a threshold at the 70.7% correct point for a theoretical observer that achieves the bound by:

$${\mathrm{threshold}}_{\theta }=\sqrt{\sigma_{\hat{\theta }}^{2}}$$ (3)

However, this solution places a strong constraint on the types of tasks that can be modeled because it assumes that the stimulus is deterministic [56]. In contrast, our behavioral tasks include random noise in the acoustic stimulus as well as randomized stimulus parameters (e.g., level, phase) that may limit an observer’s ability to discriminate changes in *θ* beyond the limits imposed by the mapping from acoustic waveform to neural response and by neural noise. Unfortunately, acoustic noise in the stimulus is not readily addressed and instead must be handled via approximations or deviations from optimality [69,70]. However, Eq 2 *can* be generalized to account for randomness in stimulus parameters in two steps. First, *θ* can be treated as a random variable rather than as an unknown constant [56,69,83]. Under suitable conditions:

$$\frac{1}{\sigma_{\hat{\theta }}^{2}}\leq E_{\theta }[I(\theta )]+A(\theta )$$ (4)

where

$$A(\theta )=-E\;\left(\frac{\partial^{2}\log p(\theta )}{\partial \theta_{2}}\right)$$ (5)

When θ ~ N(μ, ν^2^), then A(θ) = 1/ν^2^ and is equal to the Fisher information of the location parameter μ in θ. In other words, the precision of the optimal estimator of θ is bounded above by the sum of average Fisher information of θ, or E_θ_[I(θ)] and a priori information about the location of θ, or A(θ). However, this solution still constrains the types of tasks that can be modeled in that *θ* is scalar. The second step is to generalize from a scalar-valued θ to a vector-valued **θ** [83,84]. The multivariate form of Eq 4 replaces scalar-valued θ with vector-valued **θ** and scalar-valued $\sigma_{\hat{\theta }}^{2}$ with variance-covariance matrix $\mathrm{var}(\hat{\theta })$ to arrive at:

$${\mathrm{var}(\hat{\theta })}^{-1}\leq E_{\theta }[I(\theta )]+A(\theta )$$ (6)

***θ*** is an *n*-dimensional vector where *n* is the number of stimulus parameters in the discrimination task. One parameter (e.g., *θ*_1_) may be the focus of the task whereas other parameters may be “nuisance” parameters whose influence the listener is supposed to ignore. $\mathrm{var}(\hat{\theta })$ is the variance-covariance matrix of $\hat{\theta }$, the estimator of ***θ***. It can be shown that, for non-homogeneous Poisson distributions with rate parameters *r*_*i*_(*t*, *θ*), the elements of *I*(***θ***) are of the form:

$${I(\theta )}_{\alpha ,\beta }=\sum_{i}\int_{0}^{T}\frac{1}{r_{i}(t,\theta )}\frac{\partial r_{i}(t,\theta )}{\partial \alpha }\frac{\partial r_{i}(t,\theta )}{\partial \beta }dt$$ (7)

Eq 7 is the result of direct simplification from the definition of Fisher information and Eq 4. To calculate JND, we first invert both sides to arrive at:

$$\mathrm{var}(\hat{\theta })\geq {\left(E_{\theta }(I(\theta ))+A(\theta )\right)}^{-1}$$ (8)

The first element along the diagonal of $\mathrm{var}(\hat{\theta })$ in Eq 8 indicates the lower bound on the variance of any unbiased estimator of θ_1_, which can be transformed into a JND for θ_1_ as in Eq 3. Eq 8 thus allows for variations in the stimulus due to random fluctuations in stimulus parameters (e.g., level, frequency) to be properly modeled. In our use case, the expectation in Eq 8 is approximated by taking a simple mean of I(**θ**) for various sampled values of **θ**.

For the sake of demonstration, consider the two-dimensional case. Assume that ***θ*** is a two-dimensional vector, where the first element *θ*_1_ is an unknown constant to be discriminated and *θ*_2_ is a random variable with *θ*_2_ ~ *N*(*μ*, *ν*^*2*^). Then, *I*(***λ***) will be a 2×2 matrix of zeros except for the second diagonal element, which will be $\frac{1}{\nu^{2}}$ (the Fisher information for the mean [location parameter] of a normal distribution with variance *ν*^*2*^). This encodes our assumption that *θ*_1_ is a fixed (but unknown) parameter and *θ*_2_ is a random variable with a normal distribution [27]. Then, the variance of estimating *θ*_1_ is bounded by:

$${\left[\mathrm{var}(\hat{\theta })\right]}_{1,1}^{-1}\leq E_{\theta }(\sum_{i}\int_{0}^{T}\frac{1}{r_{i}(t,\theta )}{\left(\frac{\partial r_{i}(t,\theta )}{\partial \theta_{1}}\right)}^{2}dt)-\frac{{\left(E_{\theta }(\sum_{i}\int_{0}^{T}\frac{1}{r_{i}(t,\theta )}\frac{\partial r_{i}(t,\theta )}{\partial \theta_{1}}\frac{\partial r_{i}(t,\theta )}{\partial \theta_{2}})dt\right)}^{2}}{E_{\theta }(\sum_{i}\int_{0}^{T}\frac{1}{r_{i}(t,\theta )}{\left(\frac{\partial r_{i}(t,\theta )}{\partial \theta_{2}}\right)}^{2}dt)+\frac{1}{\nu^{2}}}$$ (9)

As described in Heinz et al. [56], Eq 9 admits an intuitive interpretation. The first term is the expectation of Eq 2 over *θ* and reflects changes in firing rate associated with changes in our parameter *θ*_1_. As this term grows, the variance decreases and hence the threshold improves. The second term can be thought of as a penalty against the first. The numerator of the second term goes to zero if variations in *θ*_2_ produce no change in the firing rate, and in this case the entire equation reduces to the scalar ideal observer in Eq 2. However, as variations in *θ*_2_ produce larger and larger changes in the firing rate, the second term will grow and the variance will increase, resulting in a worsening of the threshold. The impact of this term is mitigated in part by its denominator, which quantifies the extent to which the value of *θ*_2_ can be deduced from changes in firing rate and the amount of *a priori* information available about the value of *θ*_2_. For example, if *ν*^*2*^ is extremely small, suggesting that the value of *θ*_2_ does not vary much, then even if the firing rate is highly sensitive to changes in *θ*_2_ the threshold will only be minimally impacted by the second term due to the contribution of the $\frac{1}{\nu^{2}}$ in the denominator.

Eq 8 can be generalized to an arbitrary number of random continuous stimulus parameters and can model a range of paradigms [27,28,56,69,70], making it a highly useful tool for analysis of the auditory nerve in discrimination tasks. However, it still faces two primary issues that limit its general applicability. First, as was previously mentioned, it is limited in that it cannot account for the effect of acoustic noise added to the stimulus. We approximated the effect of masking noise in part by limiting the range of CFs from which information was utilized in the ideal-observer calculations (under the assumptions that CFs far from stimulus frequencies would contribute little useful information in the presence of masking noise). However, this is an incomplete account of how noise could affect optimal thresholds. Future work should seek to address this issue, possibly by developing plausible approximations or deviations from optimality [69,70]. Second, it is limited in that the Cramér-Rao lower bound does not guarantee the existence of an estimator that can achieve the bound. In other words, Eq 8 states that no system estimating ***θ*** in an unbiased fashion from auditory-nerve responses can achieve performance better than the bound but offers no insight into whether any real-life system (neural or otherwise) can instantiate an estimator that achieves the bound. Nevertheless, investigating how the lower bound varies with stimulus parameters may still provide useful insights into the link between F0 discrimination and neural coding at the earliest levels, even if no realistic estimator achieves the bound.

## Supporting information

S1 Text Auditory-nerve model validation. (DOC)

S2 Text Effects of parameter roving on ideal observer. (DOC)

S3 Text Ideal observer models at high sound levels. (DOC)

S4 Text Simulating the effects of complex-tone maskers. Fig A: Evaluating the applicability of ideal-observer analysis to complex-tone masker stimuli.
(DOC)

S1 Fig Example neurograms for all masker stimuli. Neurograms generated as in Fig 1 for the ISO, GEOM, and DBL stimuli (different rows) at low frequencies (left column) and high frequencies (right column). Simulations were conducted using medium-spontaneous-rate fibers at levels of 50 dB SPL per-component for both target and masker components. TEN was not included in the simulations.
(TIF)
