# Supplementary material for: Human discrimination and modeling of high-frequency complex tones shed light on the neural codes for pitch
Source: PLoS Comput Biol. 2022 Mar 3;18(3):e1009889. doi: 10.1371/journal.pcbi.1009889 (PMC8923464; doi:10.1371/journal.pcbi.1009889)
Supplement: S1 Text — (DOC) [file pcbi.1009889.s001.doc]

Several simulations were conducted to qualitatively validate auditory nerve model responses to high-frequency and concurrent harmonic stimuli. First, phase-locking strength was measured for high spontaneous rate model fibers responding to 350-ms pure tones with 20-ms raised-cosine ramps at a range of frequencies spaced logarithmically from 200 Hz to 20000 Hz. At each frequency, the CF of the simulated fiber was equal to the frequency of the pure tone stimulus. The level of the pure tone was set to 20 dB above this fiber’s threshold estimated using the procedure describe in the methods. Instantaneous firing rates were simulated for each auditory nerve model and then used as input to a Poisson spike generator to generate spikes 150 times. A Poisson spike generator was used instead of model-specific spike generators in order to put the different auditory nerve models on equal footing and to ensure that the phase locking estimates reflected the lowpass characteristic of the inner hair cell stage and not any effects of a model-specific spike generator (whose impacts would not be reflected in the ideal observer analysis, which only used instantaneous firing rates). Phase locking at frequency *f* was quantified in terms of vector strength *v*(*f*) [1] according to

$$\begin{aligned} v\left( f \right)=\left| \frac{1}{n}\sum_{i=1}^{n} \exp\left( j2\pi ft_{i} \right) \right|\#\left( 1 \right) \end{aligned}$$

where *t_i_* denotes the time of the *i*-th spike, *n* denotes the number of spikes, and *j* denotes the imaginary unit. The vector strength was estimated over the 150 responses to the pure tone (i.e., the sum extended from the first spike of the first simulation to the last spike of the last simulation) at each frequency for each model. This process was repeated 50 times and the vector strength curves from each repetition were averaged to arrive at final estimates. As can be seen (Fig A), in all of the tested nerve models, vector strength begins to roll off around 1-2.5 kHz, rolls off rapidly, and becomes very weak (vector strength $< 0.1$) beyond 4-5 kHz (consistent with available physiological evidence) [2,3,4]. Marked differences exist between the different auditory nerve models in terms of cutoff and slope; these differences are reflected directly in the ideal observer predictions (Figs 6 and 7).


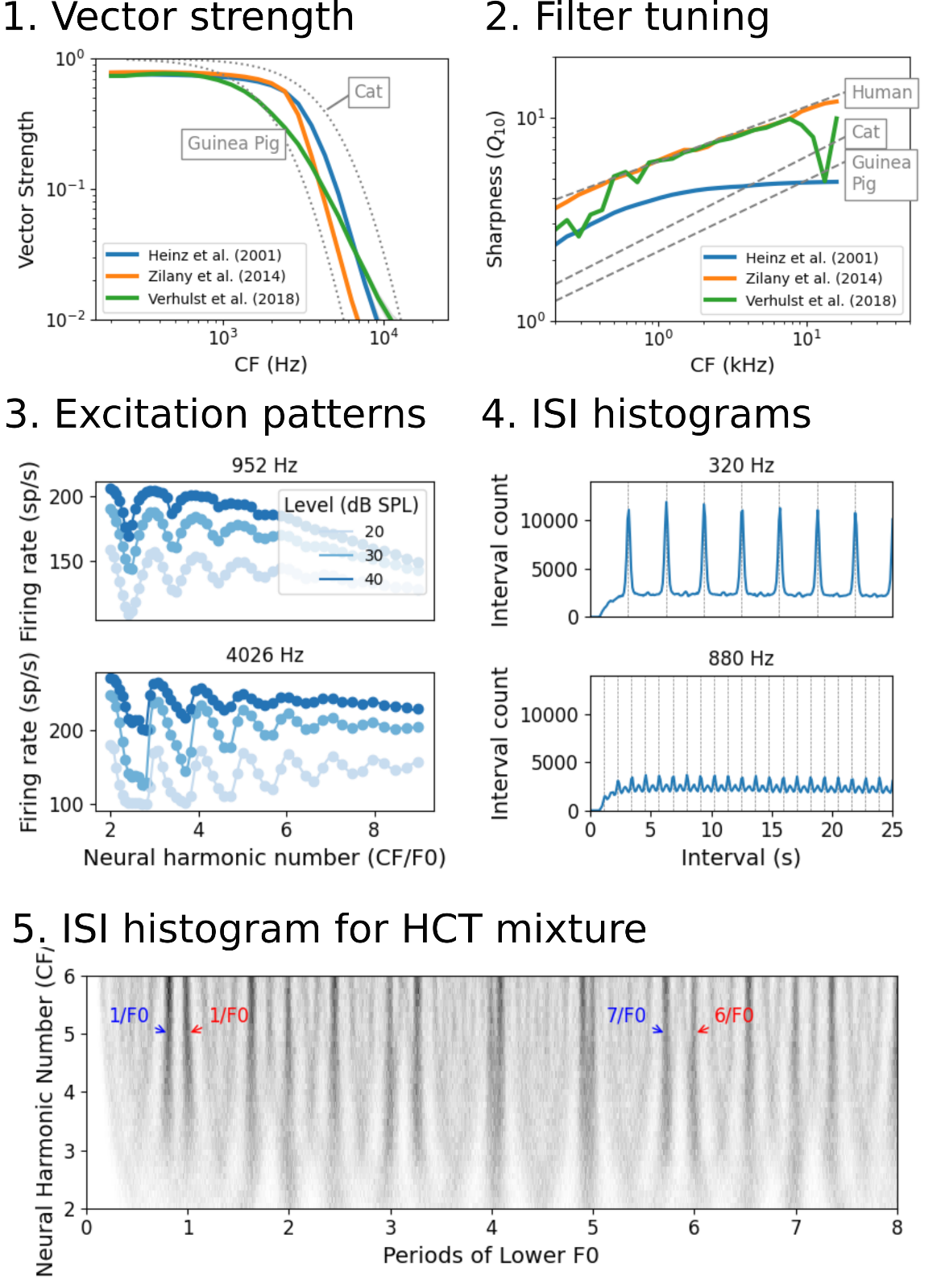


***Fig A.*** *(1) Vector strength against CF for each auditory nerve model. Colored lines indicate data from the nerve models with filled areas around the lines indicating* $\pm1$ *standard deviation, while dashed gray lines indicate data from the models of vector strength rolloff for cat and guinea pig from Weiss and Rose*


(4)*. (2) Sharpness estimates (*$Q_{10}$*) derived from iso-response tuning curves (Heinz et al. and Zilany et al. models) or basilar membrane impulse responses (Verhulst et al. model). Gray dashed lines indicate human model fits adapted from Oxenham and Shera [5] and animal model fits adapted from Shera and Guinan [6]. (3) Excitation patterns for complex tone stimuli from Cedolin and Delgutte [7] for the Zilany et al. [8] nerve model. Dots indicate the underlying data while the curves indicate a LOESS curve fit to the data. Note that the x-axis is in units of CF/F0; thus, peaks in excitation corresponding to resolved stimulus components are expected to be in the same position in the top panel (showing a lower CF of 952 Hz) and the bottom panel (showing a higher CF of 4026 Hz). (4) ISI histograms for complex tones as in Cedolin and Delgutte [7] for the Zilany et al. nerve model. Vertical dashed lines indicate the interspike intervals corresponding to the period of the F0 and integer multiples of the period of the F0. (5) Two-dimensional ISI histograms for a complex tone mixture as in Larsen et al. [9] for the Zilany et al. nerve model. The x-axis is in units of the period of the lower F0 in the complex tone mixture, the y-axis is in units of CF/F0, and color of a bin indicates the number of spikes observed in that bin (with darker colors indicating more spikes). Arrows and text labels highlight a few examples of dark bands that span vertically over a wide range of CF/F0, which indicate accurate coding of the upper F0 (blue) and lower F0 (red) over a range of harmonic numbers.*

Second, tuning bandwidths were estimate for each auditory nerve model. For the Heinz et al. [10] and Zilany et al.


[8] models, iso-response tuning curves were estimated for high spontaneous rate model fibers responding to 200 ms pure tones with 20 ms raised-cosine ramps. For a given CF, instantaneous firing rate responses were simulated for 30 tone frequencies ranging logarithmically from 0.6 octaves below CF to 0.6 octaves above CF and 30 tone levels ranging from -10 dB SPL to 40 dB SPL. These responses were then averaged over time to construct a frequency-level average firing rate profile. This profile was then upsampled by a factor of 5 using linear interpolation and an iso-response tuning curve was estimated at $1.05$ spontaneous firing rate using the marching squares algorithm. Tuning was summarized by calculating $Q_{10}$ (ratio between the CF determined from the tuning curve and the bandwidth of the tuning curve at 10 dB above the threshold) for each tuning curve. For the Verhulst et al. model


[11], computing iso-response tuning curves at the level of the auditory-nerve fibers using the same procedure was computationally prohibitive. Instead, *Q*_ERB_ values were computed from basilar membrane impulse responses. First, basilar membrane responses were simulated for an 80 microsecond click at 40 dB SPL. Next, *Q*_ERB_ values were calculated for each channel by taking the power spectrum of the response in each channel, computing the area under the curve, and dividing by the CF of the channel


[11]. These *Q*_ERB_ were converted into $Q_{10}$ values using the ratio of 1.83 suggested by Verschooten et al. [12]. As can clearly be seen (Fig A1), tuning was sharper at high CFs than at low CFs for the Zilany et al.


[8] and Verhulst et al.


[11] models (consistent with available physiological and psychophysical evidence) [4, 5, 6] but not for the Heinz et al.


[10] model.

It should be no surprise that the tested auditory-nerve models produced sensible tuning curves and phase locking curves for pure tone stimuli, as the parameters of the models are tuned to accurately recreate auditory nerve responses to such stimuli. However, our stimuli were considerably more complex than pure tones, consisting of complex tones at both low and high frequencies that were sometimes presented simultaneously with other complex tones. To ensure that the auditory-nerve model also produced sensible responses for such HCT stimuli, at least insofar as available auditory-nerve data could indicate, we synthesized subsets of the HCT stimuli used in Cedolin and Delgutte


[7] and in Larsen et al.


[9] and attempted to replicate key figures from those papers using the Zilany et al.


[8] model. First, we synthesized broadband HCTs composed of all harmonics of the F0 in cosine phase beginning with the 2nd harmonic at a level of 15 dB SPL per-component. The tones were 200 ms in duration and had 20 ms raised-cosine ramps. Tones with F0s of 320 Hz and 880 Hz (matching the examples from Fig 9 of Cedolin and Delgutte


[7]) were synthesized. Then, responses to these tones were simulated for a population of 60 fibers high spontaneous rate fibers with CFs logarithmically distributed from 450 Hz to 9200 Hz. The “cat” parameter set was used for these simulations and 100 spike trains were simulated for each fiber. All-order inter-spike interval (ISI) histograms were constructed from the simulations with a bin width of 0.5 ms. The key trends observed by Cedolin and Delgutte


[7] were captured: (1) the ISI histogram for the 320 Hz responses showed significantly greater peak-to-valley ratios and peak heights compared to the histogram for the 880 Hz responses; (2) for both histograms the interval corresponding to the F0 and its integer multiples were the most common intervals; (3) integer multiples of the F0 interval were typically as common as the F0 interval itself (Fig A4).

Next, we synthesized broadband HCTs in the same way as before and simulated average rate responses of two model auditory-nerve fibers, one with a CF of 952 Hz and one with a CF of 4026 Hz (as in Fig 4 of Cedolin and Delgutte [7]), to these such tones with a range of F0s. Average rate estimates were then plotted against neural harmonic number (i.e., CF/F0; Fig A). These simulations were conducted at stimulus levels of 20, 30, and 40 dB SPL per-component. As can be seen (Fig A3), key trends observed in the data of Cedolin and Delgutte [7] were captured qualitatively by the neural simulation. First, when the model fibers were likely unsaturated at lower stimulus levels, low-order harmonics were resolved in the average rate responses. Moreover, the lowest resolved harmonic was higher for the high CF (4026 Hz) than for the low CF (952 Hz), consistent with sharper relative frequency tuning at high CFs in the auditory nerve. Second, as the stimulus level increased the highest resolved harmonic number progressively decreased both for the low- and high-CF fibers, consistent with the idea that tuning broadens and rate-place coding worsens at higher levels. In other words, this analysis suggests that the impact of level-dependent frequency tuning of the auditory nerve on the representation of HCTs is captured reasonably well by the Zilany et al. [8] model.

Second, we synthesized mixtures of HCTs at levels of 70 dB SPL per component and simulated responses for these tones from a single medium spontaneous rate auditory-nerve fiber with a CF of 816 Hz, as in Fig 6 of Larsen et al. [9]. The HCTs were synthesized in the same way as before except that they only contained harmonics 2-20 of their F0s. The simulation was conducted for a range of F0s, with the F0 of the lower tone ranging from 136 to 408 Hz and the F0 of the higher tone always being fixed at 11/9 times the F0 of the lower tone. For each HCT mixture, 200 spike trains were simulated and then an ISI histogram was constructed with the time axis in units of the F0 period. This simulation qualitatively replicated key findings of Larsen et al. [9], in that the periods of both F0s and their integer multiples are clearly represented in the distribution of ISIs across the full range of tested F0s (Fig A5). This analysis suggests that the Zilany et al. [8] model does a good job at capturing the extent to which temporal information from mixtures of complex tones is encoded in auditory nerve spike-time patterns.

Collectively, these results suggest, at least at a qualitative level, that the tested auditory-nerve models likely provide reasonable auditory nerve responses to the types of stimuli used in our behavioral experiments. There are, however, several remaining limitations that should be kept in mind. First, the available physiological data for auditory nerve responses to mixtures of complex tones is limited and, to our knowledge, does not include tones with F0s as high as listeners heard during the behavioral experiments. Inaccuracies in the simulated auditory nerve responses could invalidate our ideal observer analysis at these extreme parameter ranges. Second, the figures are all replications of animal data and it is not clear whether the same close correspondence between model and data would hold for human data (if it were available).

**References**

1. Goldberg JM, Brown PB. Response of Binaural Neurons of Dog Superior Olivary Complex to Dichotic Tonal Stimuli: Some Physiological Mechanisms of Sound Localization. Journal of Neurophysiology. 1969;32(4):613–635.

2. Joris PX, Verschooten E. On the limit of neural phase locking to fine structure in humans. In: Moore BCJ, editor. Basic aspects of hearing. New York, NY: Springer; 2013. p. 101–108.

3. Weiss TF, Rose C. A comparison of synchronization filters in different auditory receptor organs. Hearing Research. 1988;33:175–180.

4. Shera CA, Guinan JJ, Oxenham AJ. Revised estimates of cochlear tuning from otoacoustic and behavioral measurements. Proceedings of the National Academy of Sciences. 2002;99(5):3318–3323.

5. Oxenham AJ, Shera CA. Estimates of human cochlear tuning at low levels using forward and

simultaneous masking. Journal of the Association for Research in Otolaryngology. 2003;4(4):541-54.

6. Shera CA, Guinan Jr JJ. Stimulus-frequency-emission group delay: A test of coherent reflection

filtering and a window on cochlear tuning. The Journal of the Acoustical Society of America. 2003;113(5):2762-72

7. Cedolin L, Delgutte B. Pitch of Complex Tones: Rate-Place and Interspike-Interval Representations in the Auditory Nerve. Journal of Neurophysiology. 2005;94:347–362.

8. Zilany MSA, Bruce IC, Carney LH. Updated parameters and expanded simulation options for a model of the auditory periphery. The Journal of the Acoustical Society of America. 2014;135(1):283–286.

9. Larsen E, Cedolin L, Delgutte B. Pitch Representations in the Auditory Nerve: Two Concurrent Complex Tones. Journal of Neurophysiology. 2008;100:1301–1319.

10. Heinz MG, Colburn HS, Carney LH. Evaluating Auditory Performance Limits: I. One-Parameter Discrimination Using a Computational Model for the Auditory Nerve. Neural Computation. 2001;13:2273–2316.

11. Verhulst S, Altoè A, Vasilikov V. Computational modeling of the human auditory periphery: Auditory nerve responses, evoked potentials and hearing loss. Hearing Research. 2018;360:55–75.

12. Verschooten E, Desloovere C, Joris PX. High-resolution frequency tuning but not temporal coding in the human cochlea. PLoS Biology. 2012;16(10):e2005164.
