# Supplementary material for: Human discrimination and modeling of high-frequency complex tones shed light on the neural codes for pitch
Source: PLoS Comput Biol. 2022 Mar 3;18(3):e1009889. doi: 10.1371/journal.pcbi.1009889 (PMC8923464; doi:10.1371/journal.pcbi.1009889)
Supplement: S2 Text — (DOC) [file pcbi.1009889.s002.doc]

As discussed in the Materials and Methods section, the ideal observer equations are capable of modeling tasks with randomly distributed parameters. The simulation results plotted in the main text all derive from simulations where all parameters were modeled as deterministic, but here we report the results from a set of simulations treating either the levels of stimulus components as random (“level roved”) or the starting phase of stimulus components as random (“phase randomized”) (Fig A). For the latter case, note that keeping the phase fixed is essentially assuming that the observer “knows” the starting time or phase of the pure tone stimulus [1]. We found that level roving produced virtually no change in thresholds for either the all-information observer or the rate-place observer. Phase randomization increased (worsened) thresholds for both frequency discrimination and F0 discrimination by approximately a factor of 2 at most tested frequencies, consistent with the predictions of Siebert [1].


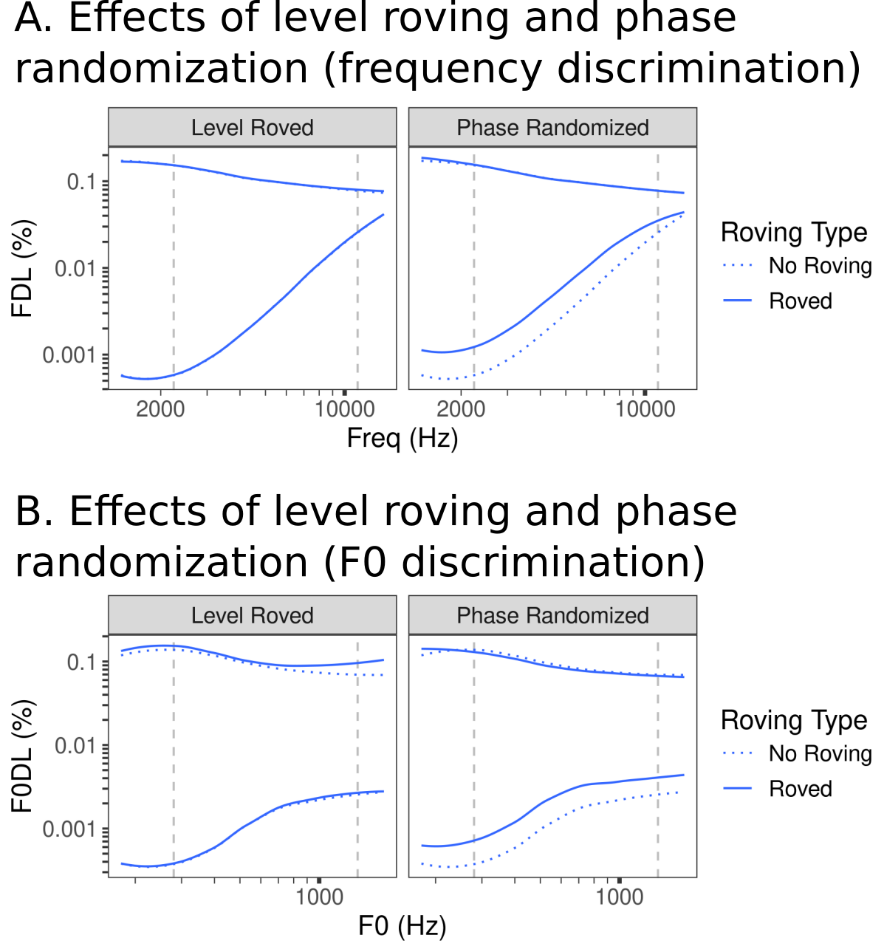


***Fig A.*** *(A) Comparison of ideal-observer thresholds with deterministic parameters versus ideal-observer thresholds with stochastic parameters for the frequency-discrimination simulations. “Level roved” refers to simulations wherein the level of the pure tone was treated as a random variable (uniform distribution over [-3, 3] dB relative to the mean of 30 dB re: threshold). “Phase randomized” refers to simulations wherein the starting phase of the pure tone was treated as a random variable (uniform distribution over [0, 360] degrees). (B) “Level roved” refers to simulations wherein the level of each component in the Experiment 1a ISO stimulus was treated as an independent random variable (uniform distribution over [-3, 3] dB relative to the nominal mean of 30 dB re: threshold). “Phase randomized” refers to simulations wherein the starting phase of each component in the Experiment 1a ISO stimulus was treated as an independent random variable (uniform distribution over [0, 360] degrees).*

**References**

1. Siebert WM. Frequency discrimination in the auditory system: Place or periodicity mechanisms? Proceedings of the IEEE. 1970;58(5):723–730.
