# Supplementary material for: Human discrimination and modeling of high-frequency complex tones shed light on the neural codes for pitch
Source: PLoS Comput Biol. 2022 Mar 3;18(3):e1009889. doi: 10.1371/journal.pcbi.1009889 (PMC8923464; doi:10.1371/journal.pcbi.1009889)
Supplement: S3 Text — (DOC) [file pcbi.1009889.s003.doc]

Most of auditory-nerve simulations in the analyses presented in the main text were conducted at low-to-moderate sound levels and at high sampling rates to avoid significant contributions from undesirable distortion. In some applications, such distortion may be of minor importance; however, for ideal-observer analysis, where the ideal observer can exploit all features of the simulated responses, such distortion can dramatically impact predictions. Significant contributions of distortion to ideal-observer predictions based on auditory-nerve model responses are previously attested in similar work using the Heinz et al. auditory-nerve model [1], and below we show evidence that distortion also impacts predictions at high levels using the Zilany et al. [2] auditory-nerve model.

However, a frequent criticism of rate-place models of pitch perception is that their performance ought to degrade significantly at higher levels (whereas human pitch discrimination changes only to a small extent with changes in level). To test whether our rate-place ideal observer demonstrated this expected breakdown at higher levels, we repeated the Zilany et al. [2] ideal-observer simulations reported in Figs 6A and 7A at higher levels than those reported in the main text (up to 80 dB re: threshold, corresponding to levels of 90+ dB SPL for all tested models). The results of these simulations are reported in Fig A.


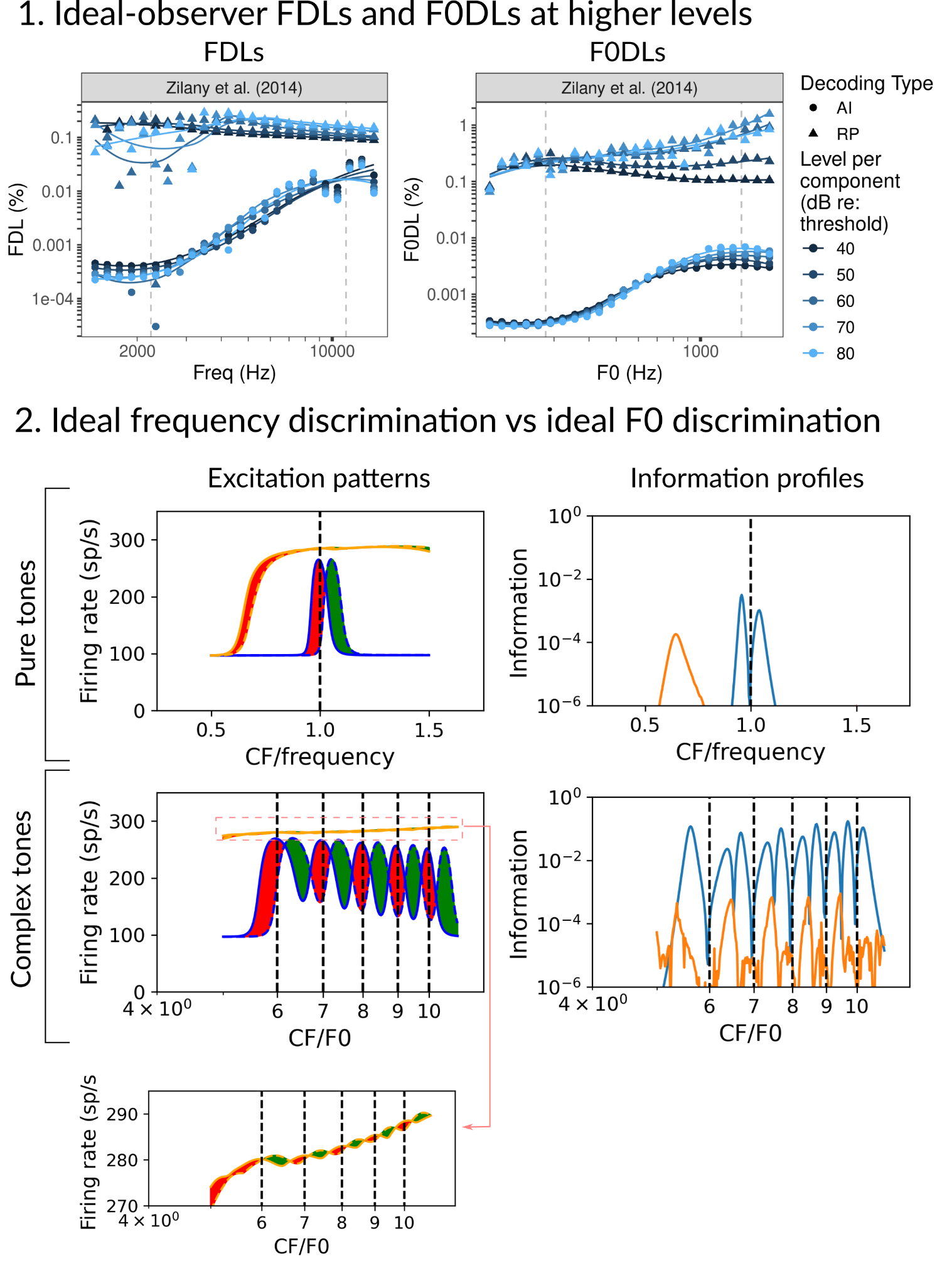
***Fig A.*** *(1) (left) Simulated FDLs versus frequency for a pure tone in the Zilany et al. [2] auditory-nerve model at a higher range of levels. Points indicate the simulated FDLs at a particular frequency while lines indicate a LOESS fit to the simulated FDLs. (right) Simulated F0DLs versus F0 for an ISO complex-tone stimulus in the Zilany et al. [2] auditory-nerve model at a higher range of levels. Points indicate the simulated F0DLs at a particular frequency while lines indicate a LOESS fit to the simulated F0DLs. (2) Simulated excitation patterns and information profiles for high-frequency pure tones and complex tones. The top row shows results for pure tones while the bottom row shows results for complex tones. In the excitation pattern column, solid lines show excitation patterns at F0 = 1400 Hz while dashed lines show excitation patterns for an F0 one semitone higher. Red and green filled regions show where the average firing rate decreases or increased, respectively, in response to the higher F0. Two sets of curves are plotted. One set is based on simulations conducted at 20 dB re: threshold (blue curves) while the other is based on simulations conducted at 80 dB re: threshold (orange curves). In the information profile column, the Fisher information at each CF (i.e., the expression inside the summation in Equation 2) is shown on a log scale. Higher values on this metric correspond to more information available to discriminate frequency (or F0) and lower discrimination thresholds.*

Consistent with data showing that auditory-nerve fibers phase lock reliably even at high stimulus level [3], changes in level over the tested range had minimal impact on all-information thresholds for both frequency discrimination (FDLs) and F0 discrimination (F0DLs). Beyond ~50 dB re: threshold, low-frequency rate-place FDLs were not stable. That is, some stimulus frequencies yielded unexpectedly good (low) thresholds. This instability was related to the presence of distorted responses to low-frequency pure tones at high levels in nerve fibers tuned to CFs below the tone frequency (Fig B). When the low-frequency side of the CF range was adjusted to exclude the response distortion and sampling rates were raised (to 500 kHz for the overall model and 250 kHz for the synapse stage), low-frequency FDLs were stable (Fig B). At higher frequencies, thresholds were stable (Fig A) and no evidence of significant distortion was observed (Fig B). Considering the results based on the adjusted CF range, increases in level up to 80 dB re: threshold produced only minor increases in FDLs at high frequencies (approximately a factor of 2 increase in thresholds from 40 to 100 dB re: threshold at *f* = 10000 Hz; S5 Fig) and somewhat larger increases in FDLs at lower frequencies (approximately a factor of 5 increase from 40 to 80 dB re: threshold at *f* = 2000 Hz; Fig B). This modest effect of level is reasonable for the present simulations, because as auditory-nerve fibers tuned near the stimulus frequency became saturated the ideal observer was still able to use information from off-CF fibers (particularly at CFs below the stimulus frequency) that were not yet completely saturated to maintain good performance at high levels (Fig A).

*
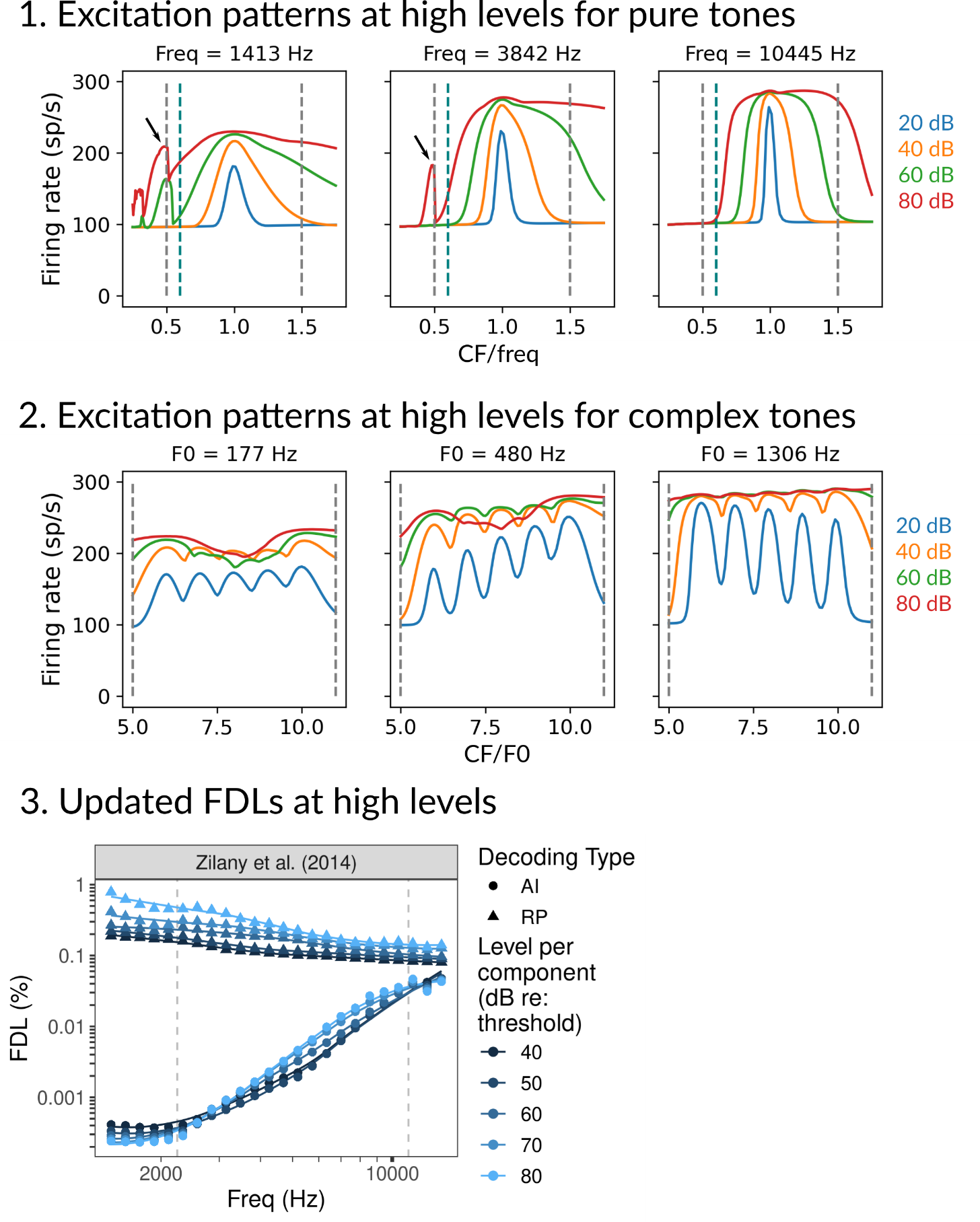
*

***Fig B:*** *(1) Excitation patterns (average firing rate versus CF) for 100 ms pure-tone stimuli at various levels (color, in dB re: threshold) and frequencies (panels). Vertical gray dashed lines indicate the extent of CFs over which the ideal observer integrated information in the main-text figures and in Fig A. The vertical blue dashed lines indicate the amount by which the lower limit of integration was increase to avoid contributions of distortion. (2) Excitation patterns for 100 ms complex-tone stimuli at various levels and F0s. The vertical dashed lines indicate the CF range over which the ideal observer integrated information. (3) Ideal-observer FDLs using a restricted range of CFs to avoid contributions from distortion and higher sampling rates (500 kHz for the overall sampling rate and 250 kHz for the synapse stage).*

In contrast, at least for F0s beyond ~400 Hz (where excitation patterns systematically flattened at higher levels instead of “bending” at higher levels; Fig B), increases in level beyond 40 dB re: threshold produced substantial increases in F0DLs for the rate-place observer (over a factor of 5 increase in thresholds from 40 to 80 dB re: threshold at F0 = 1000 Hz). Whereas for frequency discrimination the ideal observer was able to utilize information from off-CF fibers to discriminate changes in frequency at higher levels, the presence of multiple frequency components in the complex-tone stimulus meant that no unsaturated fibers at the edge of the CF range were available to robustly code F0 changes at higher levels (Fig A), resulting in poor F0 discrimination thresholds based only on very small changes in the excitation pattern.

The primary conclusion from these analyses conducted at high levels is that, as expected, rate-place observer performance degrades substantially at high levels. At sufficiently high sound levels, good performance for the rate-place observer is contingent on the presence of “off-CF” fibers that are not yet saturated. These trends are in stark contrast to the fact that humans can accurately discriminate pitch over a wide range of levels. One might rule out the rate-place observer purely on the basis that it may be outperformed by humans in some cases (e.g., some listeners in Experiment 1 achieved high-frequency F0DLs better than the rate-place observer at high levels). However, several possible factors that were not modeled here, including acoustic noise in the stimulus and the contributions of low- and medium-spontaneous-rate fibers to discrimination performance; thus, decisively ruling out the rate-place observer based on comparing its performance to human performance is premature before some of these limitations are addressed in future studies.

**References**

1. Heinz MG, Colburn HS, Carney LH. Evaluating Auditory Performance Limits: I. One-Parameter Discrimination Using a Computational Model for the Auditory Nerve. Neural Computation. 2001;13:2273–2316.

2. Zilany MSA, Bruce IC, Carney LH. Updated parameters and expanded simulation options for a model of the auditory periphery. The Journal of the Acoustical Society of America. 2014;135(1):283–286.

.3. Heil P, Peterson AJ. Spike Timing in Auditory-Nerve Fibers During Spontaneous Activity and Phase Locking. Synapse. 2017;71(1):5–36.
