# Supplementary material for: Human discrimination and modeling of high-frequency complex tones shed light on the neural codes for pitch
Source: PLoS Comput Biol. 2022 Mar 3;18(3):e1009889. doi: 10.1371/journal.pcbi.1009889 (PMC8923464; doi:10.1371/journal.pcbi.1009889)
Supplement: S4 Text — Fig A: Evaluating the applicability of ideal-observer analysis to complex-tone masker stimuli. (DOC) [file pcbi.1009889.s004.doc]

In theory, the ideal-observer equations used to analyze frequency and F0 discrimination (e.g., Eq 2) are directly extensible to complex-tone masker stimuli used in Experiment 1 (GEOM stimulus) and Experiment 2 (DBL stimulus). However, in practice, we have found that using these equations to model stimuli containing complex-tone maskers, interpreting the results, and relating the ideal-observer results to human performance is non-trivial and presents several challenges. Although a full treatment would be worthwhile, it is beyond the scope of the present paper. Nevertheless, we present here some preliminary attempts at applying these equations to stimuli with maskers. Hopefully, this initial attempt will provide a good starting point to future research on this topic.

Fig A shows the results of naively applying Eq 2 to the Experiment 1b ISO stimulus and the Experiment 1b GEOM stimulus using the Heinz et al. [1] auditory-nerve model. All stimulus and model parameters were identical to those used in the simulations shown in the main-text Figs 7 and 8, except that levels were set to 30 dB re: threshold and the interval between the target and masker in the GEOM stimulus was set to 0.5 ST. As can be seen, adding a masker 0.5 ST above the target either resulted in no change in predicted thresholds or *improvements* in predicted thresholds. At first glance, this result is somewhat surprising --- how can adding a masker *improve* estimation of the target F0? We first attempted to confirm that the result could not be explained due to inaccurate estimation of the partial derivative with respect to rate inside Eq 2. In our simulations (and, to the best of our knowledge, in similar simulations conducted in prior work), this derivative is approximated using the forward finite-differences method (Fig A). That is

$$\frac{d}{d\theta}r\left( \theta\right)\approx\frac{r\left( \theta+h \right)-r(\theta)}{h}$$

where *h* is some small constant (in units of Hz for either pure-tone frequency for FDL simulations or complex-tone F0 for F0DL simulations). The true derivative is defined as the limit of the right-hand-side as *h* goes to zero; thus, we naturally want to pick a small value for *h* to ensure that our estimate of the derivative is accurate. At the same time, we want to pick a sufficiently large value for *h* so as to avoid incurring significant inaccuracies from the limited precision of the underlying floating-point representation of the numbers. To examine the extent to which inaccuracies in this approximation might have influenced our results, we repeated the simulations in Fig A at many different values of *h* and using the backward, forward, and central finite-difference methods illustrated in Fig A. For all observers, stimuli, frequencies, and finite-difference estimation methods, simulated thresholds were stable over the range from *h* = 1e-6 to *h=*0.01 (while the simulations reported in the main text used a value of *h* = 0.001, as in [27]). At larger values of *h* (i.e., *h* > 0.01), thresholds were less stable, particularly for the masked condition. These results suggest that, at least for small values of *h* below a certain critical value, the basic derivative estimation procedure is numerically stable and accurately reflects the derivative of the underlying firing-rate function instantiated in the auditory-nerve model.


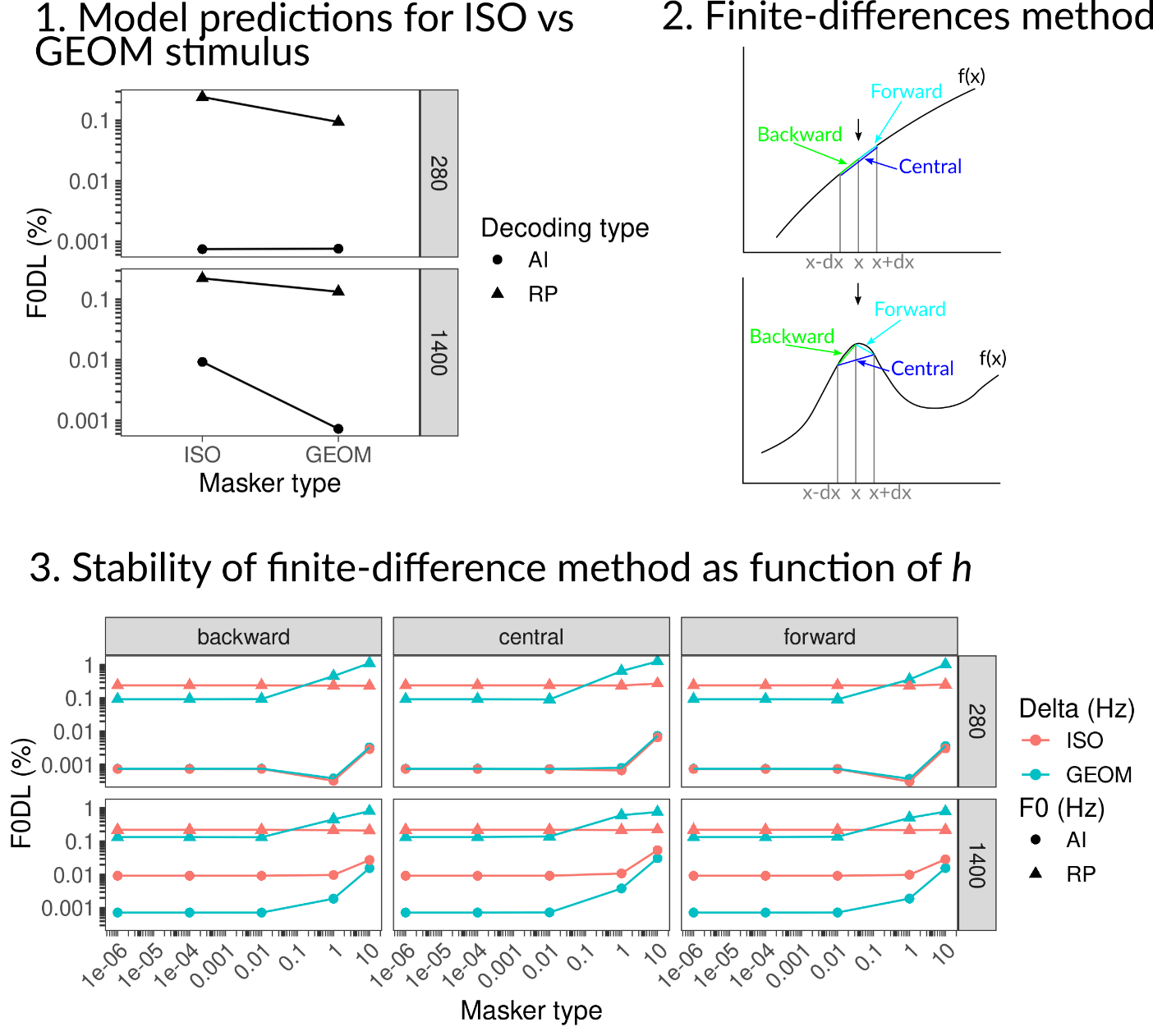


***Fig A:*** *(1) Predicted F0DLs for the Experiment 1b ISO and GEOM stimuli at low frequencies (top panel) and high frequencies (bottom panel). (2) Schematic displaying how the finite-differences method is used to estimate the derivatives in Equation 2. (3) Predicted F0DLs for the ISO and GEOM stimuli as a function of the value of h used to estimate the derivative in Equation 2 via the finite-differences method. Different panels from left to right show different tested finite-differences methods. Simulations at low frequencies are shown in the top panels while simulations at high frequencies are show in the bottom panels.*

Next, we visualized the core computation in the finite-differences approximation of the rate-place observer (i.e., difference in average firing rate for *θ* and *θ+ h* divided by *h*) as a function of *h* at a range of CFs (Fig B). The curves plotted in this figure are differences of an excitation pattern computed at a baseline F0 (*θ*) and an excitation pattern computed at an incremented F0 (*θ + h*) which is then normalized by the increment size (*h*). As can be seen, the curves for the ISO stimulus maintain their shape and magnitude as a function of *h*, consistent with the observation in Fig A that rate-place observer thresholds for the ISO stimulus are stable as a function of *h*. In contrast, the curves for the GEOM stimulus change shape substantially over the evaluated range of *h*, consistent with the observation in Fig A that rate-place observer thresholds for the GEOM stimulus are *not* stable as a function of *h*.


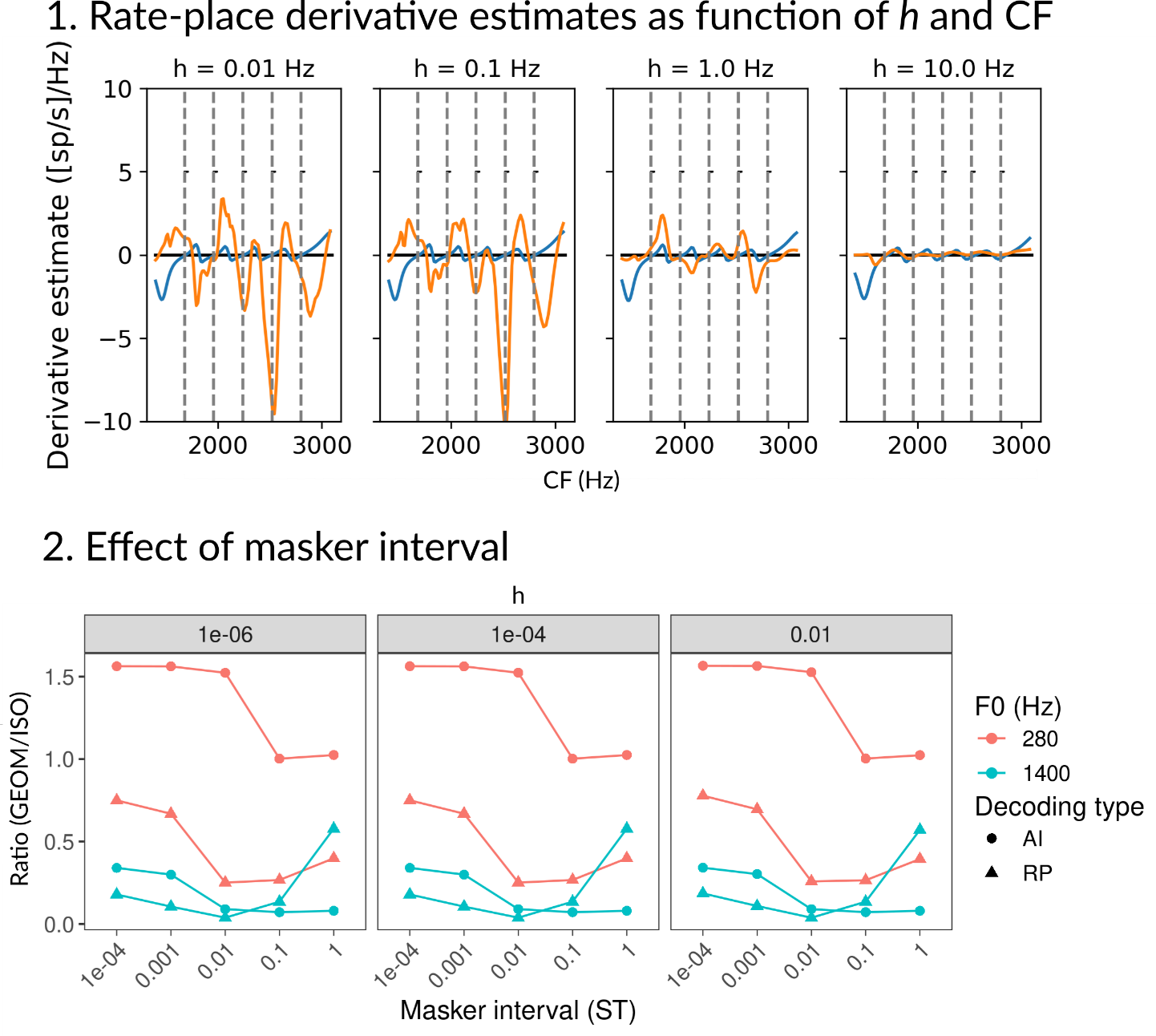


***Fig B:*** *(1) Derivative estimates for the rate-place observer (i.e., differences in excitation patterns normalized by h) as a function of CF for ISO and GEOM stimuli from Experiment 1b. Different panels show different values of h. (2) Ratios of predicted F0DLs for the GEOM stimulus vs the ISO stimulus as a function of the interval between the target and masker tones in the GEOM stimulus. Different panels show different values of h.*

One additional challenge in applying the ideal-observer equations to the masked conditions is how to set the interval between the target tone and the masker tone. The interval size that human listeners experienced was a deterministic function of the F0 difference between reference and target tones (DF0). Specifically, the masker F0 was geometrically centered between the reference and target F0s. Thus, when the adaptive procedure became more difficult and DF0 shrank, the masker interval size likewise became smaller; when the adaptive procedure became easier and DF0 grew, the masker interval size likewise became larger. It is thus not immediately clear how one should set the masker interval size in the simulation procedure. In Fig 5, we adopted the approach of selecting a moderate value for the masker interval (0.5 ST) that was of the same order of magnitude as the intervals typically heard by listeners in the behavioral experiment. However, it may be the case that a moderate masker interval does not successfully “mask” the very small value of *h* used to approximate Eq 2 in the ideal observer. To explore this possibility, we repeated the simulations from Fig A at a range of masker intervals and values for *h* (Fig B). Changes in the masker interval substantially changed the predicted thresholds, particularly for the low-frequency all-information observer; however, at almost all tested combinations of masker interval size and *h*, predicted thresholds were still *better* in the masked condition than in the unmasked condition. Another important consideration is whether to treat the masker interval as a deterministic variable or as a random variable. Eq 8 could be applied here with the masker interval treated as a random variable, as was done for level and phase in the simulations reported in S2 Text. However, again, it is not clear how the situation experienced by humans should be translated to the model equations, and the combinatoric space of possible choices to test is quite large.

To summarize, the ideal observer analyses suggest that, from a purely statistical standpoint, *more* information is available in auditory-nerve responses in the presence of a single complex-tone masker than in the absence of that masker. However, this conclusion is based measuring how auditory-nerve firing rates change with respect to very small changes in input parameters, assuming perfect knowledge of the stimulus configuration. In contrast, human listeners must maintain good performance over a range of DF0s (over the duration of the adaptive procedure) with imperfect knowledge of the stimulus configuration. One central message from the present analyses could thus be that relating ideal observers and human performance is not straightforward, and care must be taken when considering how to map a set of statistical formulae to real psychophysical procedures.

**References**

1. Heinz MG, Colburn HS, Carney LH. Evaluating Auditory Performance Limits: I. One-Parameter Discrimination Using a Computational Model for the Auditory Nerve. Neural Computation. 2001;13:2273–2316.
