# Supplementary figures and images for: Human discrimination and modeling of high-frequency complex tones shed light on the neural codes for pitch
Source: PLoS Comput Biol. 2022 Mar 3;18(3):e1009889. doi: 10.1371/journal.pcbi.1009889 (PMC8923464; doi:10.1371/journal.pcbi.1009889)

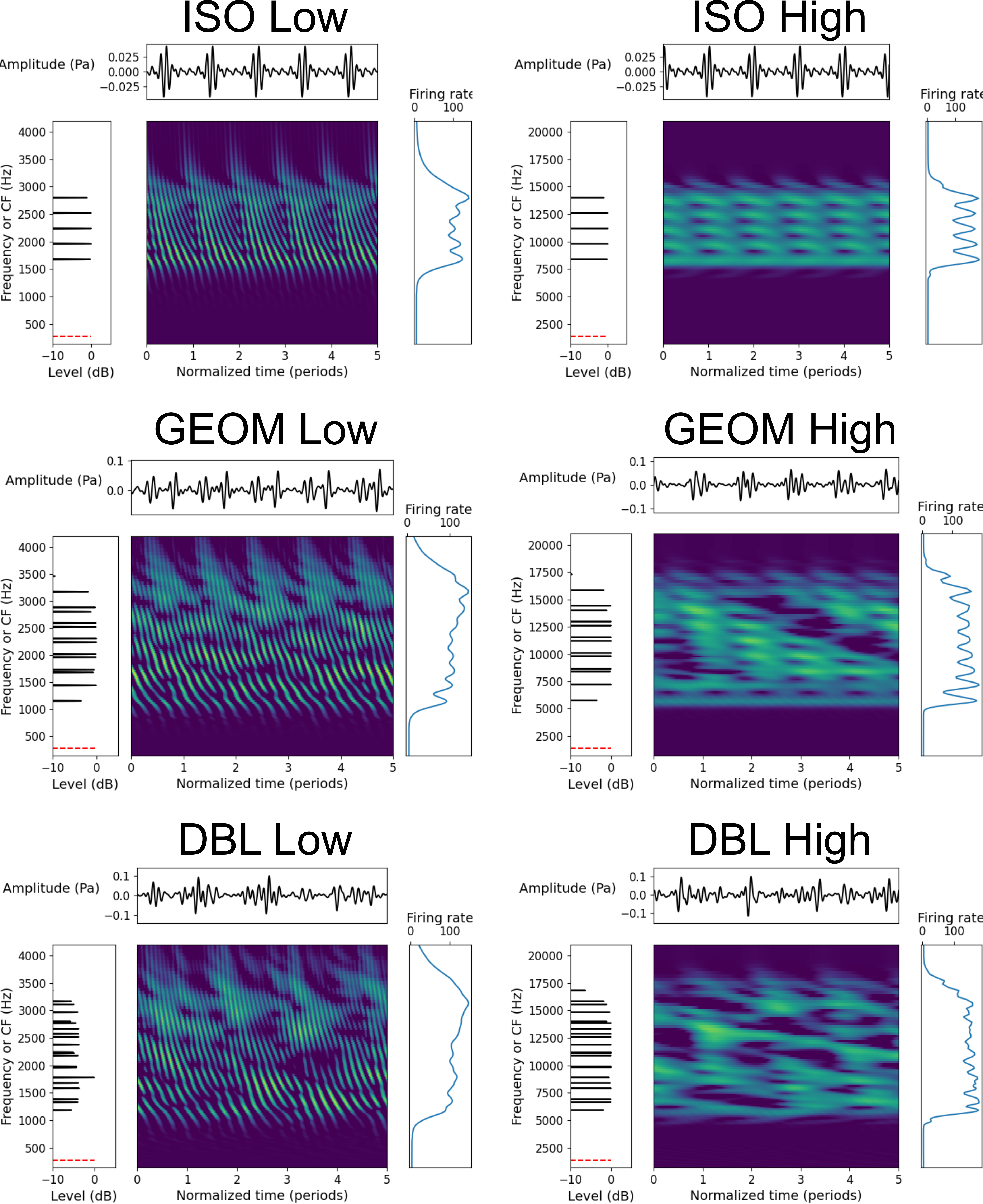

Supplement: S1 Fig — Neurograms generated as in Fig 1 for the ISO, GEOM, and DBL stimuli (different rows) at low frequencies (left column) and high frequencies (right column). Simulations were conducted using medium-spontaneous-rate fibers at levels of 50 dB SPL per-component for both target and masker components. TEN was not included in the simulations. (TIF) [file pcbi.1009889.s005.tif]
